# Microscopic crystallographic analysis of dislocations in molecular crystals

**DOI:** 10.1038/s41563-025-02138-5

**Published:** 2025-03-03

**Authors:** Sang T. Pham, Natalia Koniuch, Emily Wynne, Andy Brown, Sean M. Collins

**Affiliations:** 1 Bragg Centre for Materials Research & School of Chemical and Process Engineering, University of Leeds, Woodhouse Lane, Leeds, UK; 2 School of Chemistry, University of Leeds, Woodhouse Lane, Leeds, UK

**Keywords:** Transmission electron microscopy, Organic molecules in materials science, Structure of solids and liquids

## Abstract

Organic molecular crystals encompass a vast range of materials from pharmaceuticals to organic optoelectronics, proteins and waxes in biological and industrial settings. Crystal defects from grain boundaries to dislocations are known to play key roles in mechanisms of growth^1,2^ and in the functional properties of molecular crystals^3–5^. In contrast to the precise analysis of individual defects in metals, ceramics and inorganic semiconductors enabled by electron microscopy, substantially greater ambiguity remains in the experimental determination of individual dislocation character and slip systems in molecular materials^3^. In large part, nanoscale dislocation analysis in molecular crystals has been hindered by the low electron doses required to avoid irreversibly degrading these crystals^6^. Here we present a low-dose, single-exposure approach enabling nanometre-resolved analysis of individual dislocations in molecular crystals. We demonstrate the approach for a range of crystal types to reveal dislocation character and operative slip systems unambiguously.

## Abstract (web summary)

A low-dose, single-exposure electron diffraction approach is used to reveal the dislocation character and operative slip systems in beam-sensitive molecular crystals.

## Main

Across materials classes, dislocations in crystals have profound effects on properties—from the mechanical properties of metals^7,8^ to deleterious effects on performance in semiconductors^9^ as well as the advantageous modulation of charge transport properties in oxides^10^. These findings have relied heavily on electron microscopy analysis. In characteristically anisotropic molecular crystals, with molecules rather than atoms at the lattice sites, modelling and experiments have outlined how the structures at particular dislocation cores govern reactions in energetic materials^11^ and pharmaceutical tableting^12^, as well as producing trap states in optoelectronics^5^. Yet, there is a critical knowledge gap in analysing these dislocations at the nanoscale.

**Fig. 1:**
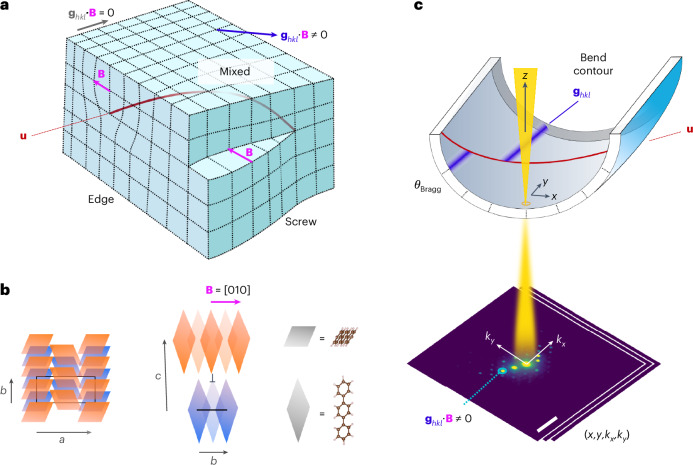
Low-dose dislocation analysis by SED. **a**, Illustration of the distortion of planes due to edge, mixed and screw dislocations, with no distortion for planes corresponding to diffraction vectors **g**_*hkl*_ at the invisibility criterion (**g**_*hkl*_ ∙ **B** = 0). **b**, Schematic of an edge dislocation core in a *p*-terphenyl crystal with Burgers vector **B** = [010]. The blue parallelograms represent molecules in a plane below those in orange. A black rectangle or line marks the *a*–*b* plane of the *p*-terphenyl lattice in the plane below (blue) the dislocation core (⊥). **c**, A bend contour appears where the sample curvature brings planes (*hkl*) to the exact Bragg condition, with displacements in the bend contour due to abrupt changes in the orientation of planes around the dislocation core, marked by the dislocation line **u**. The electron beam is scanned in (*x*, *y*), enabling the reconstruction of bend contours from multiple diffraction vectors **g**_*hkl*_ in parallel from two-dimensional diffraction patterns (*k*_*x*_, *k*_*y*_) recorded at each probe position to give a four-dimensional dataset (*x*, *y*, *k*_*x*_, *k*_*y*_). The sample bending is exaggerated for visual effect. The axes are shown as in an experiment, with the scan and diffraction cameras not generally aligned, requiring rotation calibration (Methods).

Dislocations are, in effect, a planar cut through a crystal followed by atomic displacements in a particular direction, the Burgers vector **B** (Fig. 1a). The singular displacement direction creates a linear defect, and this dislocation line vector **u** coincides with the location of maximum lattice distortion. Together, these vectors describe the operative slip systems and the character of the dislocation (that is, edge, screw or mixed). For edge dislocations, **B**⊥**u**, whereas for screw dislocations, **B**∥**u** (Fig. 1a). The classical electron microscopy approach to dislocation analysis involves the combination of diffraction and diffraction contrast imaging to evaluate the behaviour of lattice planes and associated diffraction vectors **g**_*hkl*_ in the vicinity of a dislocation. For **g**_*hkl*_ ∙ (**B** × **u**) = 0, a unique invisibility criterion is established for **g**_*hkl*_ ∙ **B** *=* 0. By exploring a range of diffraction conditions, the Burgers vector direction can be determined unambiguously.

Although scanning probe microscopy^13^, X-ray topography^14^ and modelling^3^ have supported dislocation characterization in molecular crystals, these techniques lack the spatial resolution to image individual dislocations at high density. Atomic-resolution imaging of dislocations^15^, including imaging at electron fluences of <200 e^–^ Å^−2^ in halide perovskites^16^, and three-dimensional imaging modes^17,18^ have been demonstrated, but none of the existing approaches support dislocation analysis at ~10 e^–^ Å^−2^ as required for the electron microscopy of many organic molecular crystals^6^. Moreover, the complex molecular packing of organic crystals in projection (Fig. 1b) generally precludes the lattice imaging of dislocations beyond a few high-symmetry orientations^19,20^. Early work on real-space crystallography supported the inference of plausible Burgers vector directions^21–23^, but these analyses have only been reported for molecular crystals with relatively high electron-beam stability. Our aim is to develop a low-dose method that is compatible with diverse sample preparations and supports practical application across varied instrumentation and sample environments.

Scanning electron diffraction (SED), a type of four-dimensional scanning transmission electron microscopy (4D-STEM) with a nearly parallel, low-convergence-angle probe^24^, suggests a route to low-dose electron microscopy for dislocation analysis. SED combines real- and reciprocal-space data acquisition simultaneously and can be operated at sufficiently low fluences to detect mosaicity in peptide crystals^25^ and molecular packing in organic semiconductors^26–28^. We now establish a methodology for single-exposure SED analysis of in-plane dislocations in thin, electron-transparent organic crystals at fluences as low as 5 e^–^ Å^−2^. We apply this approach to aromatic molecules, long-chain hydrocarbons and hydrogen-bonding crystals as key optoelectronic, wax and pharmaceutical models, respectively.

SED enables the parallel acquisition of many diffraction vectors by recording a two-dimensional diffraction pattern at each probe position in a scan (Fig. 1c). By selecting individual **g**_*hkl*_, we construct virtual dark-field (VDF) images. Bending of the electron-transparent crystals deposited onto electron microscopy grids results in the appearance of bands (denoted as bend contours), marking the positions in the sample that are exactly at the Bragg condition. For planes that are distorted by the dislocation (**g**_*hkl*_ ∙ **B** ≠ 0), a break in the otherwise-continuous bend contour appears at the dislocation line **u** (refs. ^21,22,29^), akin to features observed in convergent-beam electron diffraction^30^. This break corresponds to a displacement of the bend contour along **u** (Supplementary Note 1). For **g**_*hkl*_ at the invisibility criterion, the contour remains continuous. By contrast, planar and point defects would exhibit a change in all **g**_*hkl*_ values or, if correlated and symmetry breaking, would introduce additional **g**_*hkl*_.

In SED, all the recorded **g**_*hkl*_ values in the diffraction plane of sufficient intensity can be used to construct VDF images for analysis as a function of the azimuthal angle in the diffraction plane *φ* (Supplementary Fig. 1). A geometric model of the tilt of lattice planes across the dislocation core describes the angle-dependent contour displacement *f*(*φ*) for in-plane dislocations (Supplementary Figs. 2–5):

1 $$f\left(\varphi \right)=A\arctan \left(B{\cos }^{2}(\varphi -C)\right),$$

where *A*, *B* and *C* are the fitting coefficients related to the effective radius of curvature, Burgers vector and azimuthal orientation of the diffraction pattern on the detector, respectively. The displacement is zero at the invisibility criterion and maximum along the Burgers vector. The crystal orientation given by the diffraction pattern together with the Burgers vector direction (equation (1)) provides the necessary information to identify the operative slip system (Supplementary Note 1), as well as the dislocation character by inspection of the relative directions of **B** and **u**.

**Fig. 2:**
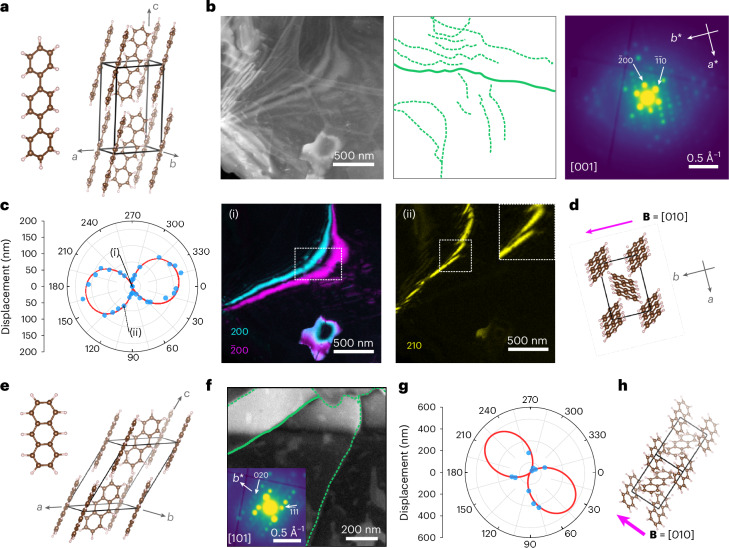
Dislocation analysis in organic optoelectronic materials. **a**, *p*-Terphenyl molecule and *p*-terphenyl unit cell. **b**, ADF-STEM image, dislocation network (green lines tracing the diffraction contrast features of dislocations) and average diffraction pattern extracted from a single SED (4D-STEM) dataset. The diffraction pattern indexes to the [001] zone axis. **c**, Polar plot of the bend contour displacements at the solid dislocation line highlighted in **b** for a series of **g**_*hkl*_ values as a function of *φ*. The red trace marks a fit to equation (1), with the measured displacements in blue. Two VDF images corresponding to the displacements marked as (i) and (ii) depicting bend contours at the **g**_*hkl*_ ∙ **B** = 0 and **g**_*hkl*_ ∙ **B** ≠ 0 conditions, respectively. The indices *hkl* are marked on the individual VDF images. **d**, *p*-Terphenyl unit cell oriented to match the sample orientation. The magenta arrow marks the Burgers vector **B** = [010]. **e**, Anthracene molecule and unit cell. **f**, ADF-STEM image and the overlaid dislocation network extracted from the image contrast. The image in the inset shows the average diffraction pattern of the entire field of view indexed to the [101] zone axis. **g**, Polar plot of the bend contour displacement as a function of *φ* for the solid green dislocation line highlighted in **f**. The red trace marks a fit to equation (1) with the measured displacements in blue. **h**, Anthracene unit cell oriented to match the sample orientation. The magenta arrow marks the Burgers vector **B** = [010]. Source data

Figure 2 presents a first demonstration applied to *p*-terphenyl (C_18_H_14_; CCDC 1269381) and anthracene (C_14_H_10;_ CCDC 1269381). Both crystallize in the *P*2_1_/*a* space group with herringbone packing. Although *p*-terphenyl is a relatively stable benchmark organic crystal^22^ (with a critical fluence (CF) > 330 e^–^ Å^−2^; Supplementary Fig. 6), anthracene is not (CF ≈ 20 e^–^ Å^−2^; Supplementary Fig. 7). Figure 2b shows an annular dark-field (ADF) STEM image of a *p*-terphenyl film with a dense dislocation network retrieved from multiple VDF images (Methods). The length of individual dislocations, for example, >2 μm, indicates that they are predominantly in plane (<3° out of plane for film thicknesses of 10–100 nm). The dislocation density is 0.32–3.2 × 10^10 ^cm^−2^ (Supplementary Table 1). The area-averaged diffraction pattern was indexed to [001], as expected^22^. Figure 2c presents the recorded bend contour displacements as a function of azimuthal angle *φ* (relative to the horizontal axis of the diffraction pattern; Supplementary Note 1) in a polar plot. A fit to equation (1) is overlaid. Supplementary Note 2, Supplementary Figs. 8 and 9 and Supplementary Table 2 provide details of the curve-fitting process and parameters. Although sizable displacements are visible for **g**_*hkl*_, for example, **g**_210_ (*d* spacing, 3.28 Å), none of them are visible for **g**_200_ or ${{\bf{g}}}_{\bar{2}00}$ (*d* spacing, 4.05 Å) (Fig. 2c). On the basis of the in-plane assumption, we show that **B** = [010] (equation (6) in the Supplementary Information), with mixed dislocation character; here the operative slip system is identified as [010](001), again as expected^22^. Supplementary Fig. 10 shows two further dislocations from the same area, similarly assigned as mixed, **B** = [010] dislocations.

Critically, the fitting approach (equation (1)) means that SED-based dislocation analysis is amenable even for a few **g**_*hkl*_ values, for example, for a limited area with few bend contours or crystals tilted off high-symmetry zone axis orientations (Supplementary Figs. 11–14). In *p*-terphenyl, **g**_*hkl*_ > 1 Å^−1^ was recorded, presenting many spacings for identifying contours (Supplementary Fig. 13). Figure 2f presents an ADF-STEM image containing a broad bend contour (top) and corresponding dislocation network in anthracene (confirmed by VDF and diffraction pattern inspection; Supplementary Figs. 15–18). No single VDF showed a zero-displacement bend contour (**g**_*hkl*_ ∙ **B** = 0). Nevertheless, fitting of the polar plot is unambiguous (Fig. 2g) and supports the determination of **B** = [010] for this mixed-type dislocation (Fig. 2h). Not all analyses are sufficiently constrained for Burgers vector determination, but dislocation character and handedness can be retrieved and combined with the knowledge of molecular packing to produce useful analyses (Supplementary Figs. 19 and 20). The analysis of dislocations in spin-coated anthracene films demonstrates the success of the approach for varied sample preparations (Supplementary Fig. 21).

**Fig. 3:**
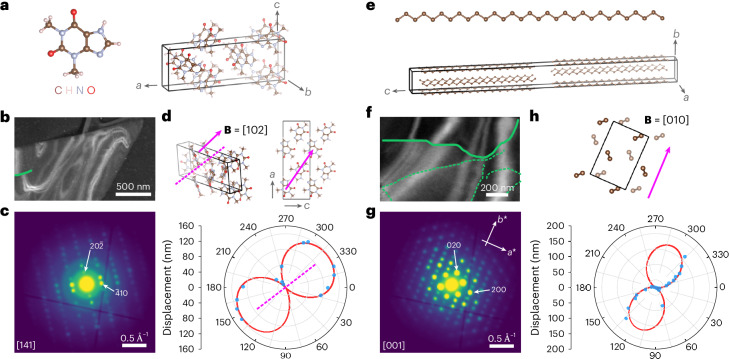
Low-dose dislocation analysis in theophylline and paraffin crystals. **a**, Theophylline molecule and unit cell of form II. **b**, ADF-STEM image with a single dislocation line overlaid in green. **c**, Corresponding electron diffraction pattern, indexed to the [141] zone axis orientation. **d**, Burgers vector determination by fitting a polar plot of the bend contour displacement as a function of *φ* to equation (1) for the dislocation line highlighted in **b**. The measured displacements are shown in blue, with the fit shown in red. A dashed magenta line marks the experimentally determined Burgers vector direction ${\bf{B}}=[1\bar{16}4]$, which aligns in projection to within ~3° with the assigned vector (**B** = [102]). The unit cell is depicted at the same orientation as given by the diffraction pattern in **c** as well as along the [010] direction to better visualize the assigned Burgers vector. **e**, *n*-Hentriacontane (C_31_H_64_) molecule and unit cell. **f**, ADF-STEM image with dislocation lines marked in green. **g**, Corresponding electron diffraction pattern, indexed to the [001] zone axis orientation. **h**, Burgers vector determination by fitting a polar plot of the bend contour displacement as a function of *φ* to equation (1) for the dislocation line highlighted in **f**. The measured displacements are shown in blue, with the fit shown in red. The unit cell is depicted at the same orientation as given by the diffraction pattern in **g**. The magenta arrow follows the long axis of the lobes in the polar plot, giving the Burgers vector direction as **B** = [010]. Source data

The versatility of our approach is further demonstrated in crystal plates (third sample preparation; Fig. 3) of the model pharmaceutical compound theophylline (C_7_H_8_N_4_O_2_; CF ≈ 26–36 e^–^ Å^−2^ for the stable form II (ref. ^6^)), and a paraffin *n*-hentriacontane (C_31_H_64_; measured CF ≈ 6 e^–^ Å^−2^, similar to other paraffins^19^). Their defects offer insights into hydration/rehydration pathways on drug storage^31^ and diffusion pathways in leaf waxes^32^. Theophylline exhibits a number of polymorphs and hydrates, and we first show an example from theophylline anhydrous form II (Fig. 3a–d). The average diffraction pattern was indexed to [141] (Fig. 3c). The dislocation is short, suggesting that the dislocation line is not in plane, testing our model’s generalization. Still, VDF analysis of the available **g**_*hkl*_ values enables fitting to equation (1) (Fig. 3d), and the geometrically recovered **B** aligns with **B** = [102] in projection, exhibiting mixed character. Additional screw-type dislocations with **B** along 〈100〉 were identified in the monohydrate form (form M; Supplementary Fig. 22) and metastable anhydrous form III (Supplementary Figs. 23 and 24). Given the differences in hydrogen bonding in the resulting dislocation cores, these dislocations may influence the dehydration pathway from form M to form II (ref. ^33^).

Figure 3e depicts the *n*-hentriacontane unit cell^34^. The plate crystal in Fig. 3f was oriented along the [001] zone axis (Fig. 3g). Here the dislocation was unambiguously identified with the Burgers vector direction along **B** = [010]. The dislocation line illustrates a full range of edge, screw and mixed character along its curved path, with the length of the dislocation line suggesting that the dislocation lies along (001), adding to reported screw dislocations^3^. Supplementary Fig. 25 presents additional pure screw dislocations in wax crystals with equivalent Burgers vectors. Going beyond edge dislocations imaged in helium-cooled paraffins^19^, in-plane mixed and screw dislocations can now be identified.

**Fig. 4:**
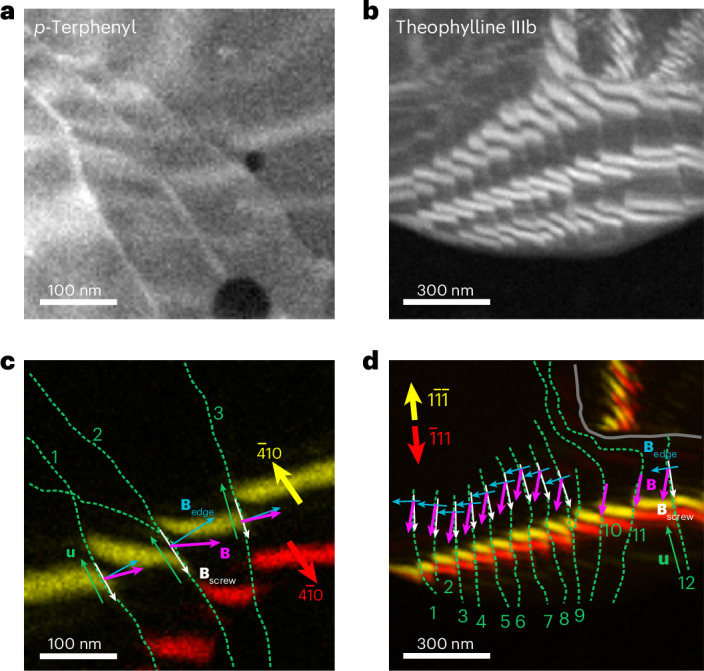
Sign analysis of interacting dislocations. **a**,**b**, Summed VDF images displaying bend contours crossing dislocation networks in *p*-terphenyl (**a**) and theophylline form IIIb (**b**). **c**,**d**, VDF images showing the selected pairs of bend contours at scattering vectors **g**_*hkl*_ that are parallel or nearly parallel to the dislocation lines, that is, along the screw component of the Burgers vector, for *p*-terphenyl (**c**) and theophylline form IIIb (**d**). The green dashed lines mark the dislocation cores (lines **u**), and the red and yellow arrows indicate the scattering vectors **g**_*hkl*_. The grey solid line in **d** outlines a grain boundary. The white arrows mark the screw components determined using the Cherns and Preston rules^30^. The magenta arrows mark the full Burgers vectors and the blue arrows mark the edge components, determined by geometric self-consistency with each screw component and Burgers vector direction from the **g**_*hkl*_ ∙ **B**_screw_ = 0 analysis (Supplementary Fig. 19). Source data

VDF images additionally support the analysis of the ‘twisting’ of bend contours at dislocations, a marker of the handedness of the screw component^29,30^ (Supplementary Note 3 and Supplementary Fig. 19). By combining the total Burgers vector direction (180° ambiguity) and the handedness for screw and mixed dislocations, we identify the handedness and the edge-component direction. Figure 4 demonstrates this approach for *p*-terphenyl and theophylline form IIIb crystals, depicting networks with aligned screw components, consistent with repulsion between such interacting screw components. Supplementary Figs. 20, 22, 24 and 25 present additional examples from anthracene, theophylline forms M and IIIb and wax crystals, respectively, also pointing to features that may pin the dislocations of opposite handedness and inviting experimentally constrained evaluation of dislocation dynamics at densities only accessible with nanoscale probes. In specific cases, the magnitude of the Burgers vector can also be determined using the established relationship^29,30^ **g**_*hkl*_ ∙ **B** = *n* (Supplementary Figs. 22–24).

In developing this approach, we also note some limitations. Measuring displacements depends on the precision in locating the centre of the bend contour. Consequently, samples containing multiple finely spaced dislocations (Supplementary Figs. 26 and 27) present challenges at finite resolution or where broad or tortuous bend contours preclude precise contour-centre localization. Excluding unusable **g**_*hkl*_ values is possible for fitting such cases. Further extensions may also be possible by analysing contours crossing several dislocations, though prior knowledge of contributing Burgers vectors may be required, and we cannot rule out deviations from linear, elastic behaviour in such regions. During beam rastering, slight reorientation of the sample can occur, which may result in shifts in bend contours mid-scan. However, these features are readily distinguished by their uniform behaviour for all **g**_*hkl*_ values (Supplementary Fig. 28). In more beam-tolerant systems (or where sample cooling supports multiple exposures), controlled tilting strategies may support Burgers vector determination from non-coplanar **g**_*hkl*_. Similarly, where sample preparation can be adjusted, controlling the curvature may support the analysis of additional model parameters. Acceleration of data processing would further enable selective measurements from pristine adjacent areas for targeted contour evaluation.

In summary, we have demonstrated in-plane dislocation analysis in varied organic molecular crystals using low-dose, single-exposure SED measurements. Introducing a geometric model for fitting, although imposing limited assumptions on slowly varying curvature, and component sign analysis enables the determination of Burgers vectors with no presumed knowledge of the crystal structure and from highly limited sampling of **g**_*hkl*_. Observed dislocations exhibit Burgers vector directions that lie predominantly in planes containing weak intermolecular interactions between layers of molecules without cutting across the molecular structure—structures that are distinct from inorganic solids. The generalization of this approach provides a means for the routine analysis of dislocations in molecular crystals and other materials with CFs down to a few e^–^ Å^−2^ (for example, using bend contours in framework materials^35,36^ and halide perovskites^37^), driving forward research into which intermolecular interactions at a dislocation core (dictated by the Burgers vector) are active in mechanical, optoelectronic and chemical properties and the development of methods for inhibiting or designing dislocation formation.

## Methods

### Sample preparation

Thin films of *p*-terphenyl and anthracene were prepared by solvent evaporation from xylene solutions^22^. Briefly, *p*-terphenyl (99.5%, Merck) or anthracene (99.0%, Merck) powder was dissolved in xylene to obtain a clear solution (~0.45–0.50 mg ml^−1^). Here 0.4 ml of the xylene solution was left to evaporate slowly on the surface of deionized water in a glass dish covered with Al foil to slow the rate of evaporation. Single-crystal films were obtained after 2 days of evaporation followed by half a day of aging. Interference colours were visible indicators of crystal thickness. Films that appeared blue or gold were sufficiently thin (120–200 nm) for electron microscopy^38^, and these films were selected for the SED experiments. The single-crystal films were transferred onto lacey-carbon-film transmission electron microscopy (TEM) support grids (EM Resolutions) by placing the grid under the film and lifting to catch the film on the TEM grid. Before the transfer process, the TEM grids were treated with an oxygen–argon (25:75) plasma for 10 s using an HPT-100 plasma treatment system (Henniker Plasma). Grids were allowed to dry in ambient air. Spin-coated anthracene films were prepared on SiN_*x*_-membrane-window TEM grids (3 × 3 SiN_*x*_ windows and 30 nm thickness; Norcada) exposed to an argon plasma for a minute. For spin coating, a 5–6 mg ml^−1^ solution of anthracene in chloroform was used. A SiN_*x*_ membrane window was fixed on a custom chuck using tape and 30 μl of the casting solution was dropped on the membrane window at 200 r.p.m. for 10 s. The spinning was then increased to 3,000 r.p.m. and the film was spun for a further minute. Finally, the SiN_*x*_ grid was annealed at 80 °C on a hot plate for 15 min.

Single-crystal films of theophylline were prepared by the solvent evaporation approach^39,40^. Briefly, theophylline powder was dissolved in nitromethane followed by heating to ~60 °C to obtain a saturated solution. The solution was then drop cast onto a holey-carbon-film TEM support grid (EM Resolutions) and left to evaporate. Highly faulted particles of partially hydrated form II plates were produced by immersion in water. A sample of theophylline monohydrate (form M) was prepared by evaporative crystallization from either ethanol:water or nitromethane:water solutions at 3:2 molar ratio followed by heating to ~55 °C. The solution was allowed to cool slowly to ~35 °C under continuous stirring for a period of 3 h. The solution was then drop cast onto a holey-carbon-film TEM support grid and left to evaporate. Form III (indexed to the form IIIb structure^33^) was identified on a TEM grid with theophylline form M (recrystallized from ethanol:water) and then immersed in liquid nitrogen. Data on form IIIb were similarly acquired under liquid-nitrogen cooling (<110 K) using a Fischione cryo-transfer holder. All other reported data were acquired under ambient conditions. Single-crystal films of *n*-hentriacontane and 1-triacontanol were similarly prepared by solvent evaporation^41^ by drop casting a supersaturated hexane solution (2 μl) onto a continuous-carbon-film TEM support grid (EM Resolutions).

### TEM

Electron diffraction pattern series as a function of cumulative electron fluence for *p*-terphenyl and anthracene were recorded using an FEI Titan^3^ Themis 300 (X-FEG high-brightness electron source, operated at 300 kV) microscope in a standard parallel-beam TEM configuration (Supplementary Note 4). For *p*-terphenyl, an electron-beam flux of 2.5 e^–^ Å^−2^ s^−1^ was used, whereas for anthracene, an electron-beam flux of 0.085 e^–^ Å^−2^ s^−1^ was used. These values were determined from the current read-out at the viewing screen, calibrated using a Faraday cup. A time series of the selected-area electron diffraction patterns were recorded for each compound across multiple crystals and multiple areas on the TEM grid.

### SED

SED data were acquired on a JEOL ARM300CF instrument (electron Physical Sciences Imaging Centre, Diamond Light Source) equipped with a high-resolution pole piece, a cold field emission gun, aberration correctors in both probe-forming and image-forming optics, and a four-chip Merlin/Medipix pixelated electron-counting STEM detector. The instrument was operated at 300 kV. Selected samples were analysed at 200 kV (identified in the corresponding figure captions). The electron optics were configured for nanobeam diffraction by switching off the aberration corrector in the probe-forming optics and adjusting the condenser lens system to produce a convergence semiangle of 0.8 mrad using a 10 μm condenser aperture. At 300 kV, this produces a 3 nm diffraction-limited probe diameter *d*_diff_ = 1.22*λ*/*α*, where *λ* is the electron de Broglie wavelength and *α* is the convergence semiangle. The probe current was measured using a Faraday cup as ~1 pA, and the exposure time at each probe position was set as 1 ms. The electron fluence was approximately 8.8 e^–^ Å^−2^ at 300 kV or 5.4 e^–^ Å^−2^ at 200 kV in a single scan, assuming a disc-like probe with a diameter equal to *d*_diff_. All the SED measurements were conducted over a scan size of 256 × 256 probe positions. Image and diffraction calibration data, including reference calibration of residual elliptical distortion in the diffraction plane, were acquired using a gold diffraction cross-grating with a period of 500 nm (Ted Pella). These cross-gratings contain a small fraction of Pd (AuPd); compared with an evaporated Au sample, we estimate ~1% error in Å^−1^ per pixel calibrations at the camera lengths used in this work when calibrating with respect to the pure Au crystal structure (with no effect on elliptical distortion calibration). To minimize these effects on our analysis, we used full pattern matching for indexation (see the ‘Data analysis’ section). The relative rotation between the diffraction pattern and raster pattern was calibrated using standard MoO_3_ crystals (Agar Scientific). Calibration data were acquired under identical conditions as the molecular crystal samples. When accounting for this calibration, the diffraction patterns are rotated, resulting in the rotation of a ‘cross’ arising from a gap in the pixel readouts between the four quadrants of the Merlin/Medipix detector.

### Data analysis

SED data were processed, aligned and calibrated using Pyxem-0.11.0 and supporting tools from the HyperSpy package (v. 1.6.5)^42^ according to previously reported procedures^43^. The direct beam in each diffraction pattern was aligned and moved to the pattern centre using a cross-correlation function in Pyxem followed by the application of an affine transformation matrix to correct the elliptical distortion and elimination of the rotation offset between the SED raster pattern and diffraction data. In some datasets, the orientation of the cross pattern (arising from the gap in pixel read-out between quadrants of the Merlin/Medipix detector) varies because the beam was positioned on different quadrants across different experiments. The ADF images were formed by integrating the diffraction pattern at each probe position between an inner radius of 0.12 Å^−1^ (2*θ* = 2.7 mrad) and an outer radius of 1 Å^−1^ (2*θ* = 22.4 mrad) to produce an image dominated by diffraction contrast. Average electron diffraction patterns were obtained by taking the mean intensity from all the diffraction patterns contributing to an image. Diffraction patterns are presented as the square root of the recorded intensity (applied in ImageJ software, v. 2.16.0) for an improved visualization of the low- and high-intensity features.

Diffraction patterns were indexed using CrystalMaker software (v. 10.7.3). The simulated kinematical electron diffraction patterns were generated from the CIF files for *p*-terphenyl (CCDC 1269381), anthracene (CCDC 1103062), theophylline (CCDC 128707 for form II, CCDC 183780 for form M and ref. ^33^ for metastable form IIIb). A unit cell for *n*-hentriacontane (C_31_H_64_) was constructed from previously reported odd-number, long-chain hydrocarbons^34^. The *Pnam* polyethylene unit cell was used for indexing the diffraction patterns from 1-triacontanol wax crystals, following the established polyethylene-like packing known for long-chain hydrocarbons^44^. Diffraction patterns at different major orientations for each material were compared with the calibrated experimental diffraction patterns in overlay. In most cases, the samples were indexed to low-index zone axes such as [001] for *p*-terphenyl, [101] for anthracene and [001] for *n*-hentriacontane and 1-triacontanol. Mis-tilt from high-symmetry zone axes (Supplementary Fig. 12) was estimated by the inspection of simulated diffraction patterns tilted away from the high-symmetry zone axis to match the experimental data. For theophylline, auto-indexing in CrystalMaker was used to assist in finding the possible zone axes as these samples could not be indexed to low-index zone axes. All the simulated diffraction patterns at the candidate zone axes were inspected and compared with the calibrated experimental diffraction patterns. Following this approach, we indexed the theophylline crystals to the zone axes [141] for form II theophylline, [$\bar{2}$13] for form M theophylline and [211] for metastable form IIIb.

Automated constructions of multiple VDF images were scripted with Python using functions from Pyxem-0.11.0 and HyperSpy-1.6.5 packages. Diffraction patterns, originally 515 × 515 pixels, were cropped and then binned by a factor of two to produce diffraction patterns with dimensions of 257 × 257 pixels. Bragg discs were detected using a peak-finding function using the difference of Gaussians method. Filter settings were tuned iteratively and assessed by manual inspection to capture all disc-like diffraction features in a randomized sub-sample of diffraction patterns. After peak finding, the position of each Bragg disc in the diffraction pattern was recorded in the coordinates of the diffraction vector (*k*_*x*_, *k*_*y*_). The peak position was further refined using a local centre-of-mass search. VDF images were then constructed from the integrated intensity within a circular aperture centred at the peak position for each probe position in the SED scan. The aperture radius was adjusted to minimize Bragg-disc overlap, typically set in the range of 0.016–0.026 Å^−1^. The corresponding diameters (0.032–0.052 Å^−1^) were fixed to be slightly smaller than the diffraction-disc diameter of 2*α*/*λ* = 0.08 Å^−1^ (at 300 kV) to limit the intensity from any adjacent diffraction spots in large unit cells with finely spaced diffraction spots (otherwise advantageous for accessing many bend contours). For a given spot at coordinate (*k*_*x*_, *k*_*y*_), the angle of the associated diffraction vector **g**_hkl_ for each Bragg disc taken as the inverse tangent of *k*_*y*_/*k*_*x*_. Dislocation lines (obscured in annular integration) were mapped by examining multiple VDF images constructed from the **g**_*hkl*_ values at which **g**_*hkl*_ ∙ **B** ≠ 0, that is, where the displacement of the bend contours is recorded. Under such conditions, the dislocation is readily traced between successive bend contour displacements across the field of view.

Bend contour displacements were measured using ImageJ software. The displacement of the bend contours on crossing the dislocation line is determined as the distance, tracing along the dislocation line, between the middle of the two bend contours (Supplementary Fig. 1 (red dashed lines) and Supplementary Note 1). The azimuthal angular variation in bend contour displacements was analysed by nonlinear least squares curve fitting, following equation (1). Curve fitting was implemented using the curve_fit function in the SciPy Python package with further evaluation of confidence intervals using the Lmfit-1.2.1 Python package^45^ (Supplementary Note 2).

## Online content

Any methods, additional references, Nature Portfolio reporting summaries, source data, extended data, supplementary information, acknowledgements, peer review information; details of author contributions and competing interests; and statements of data and code availability are available at 10.1038/s41563-025-02138-5.

## Supplementary information

Supplementary Information Supplementary Notes 1–4, Figs. 1–28 and Tables 1 and 2.

## Source data

Source Data Fig. 2 Stacked images and diffraction patterns (Fig. 2a) for *p*-terphenyl and anthracene shown in Fig. 2b,c,f.

Source Data Fig. 2 Excel file containing numerical data in polar plots for *p*-terphenyl and anthracene shown in Fig. 2c,g.

Source Data Fig. 3 Stacked images and diffraction patterns for theophylline and wax crystals shown in Fig. 3b,c,f,g.

Source Data Fig. 3 Excel file containing numerical data in polar plots for theophylline and wax crystals shown in Fig. 3d,h.

Source Data Fig. 4 Stacked images and diffraction patterns for *p*-terphenyl and theophylline crystals shown in Fig. 4.

## Data Availability

The data associated with this Article and its Supplementary Information are openly available from the University of Leeds Data Repository at 10.5518/1589 (ref. ^46^). Source data are provided with this paper.
