# Supplementary material for: Microscopic crystallographic analysis of dislocations in molecular crystals
Source: Nat Mater. 2025 Mar 3;24(5):682–7. doi: 10.1038/s41563-025-02138-5 (PMC12048348; doi:10.1038/s41563-025-02138-5)
Supplement: Supplementary file 1 — Supplementary Notes 1–4, Figs. 1–28 and Tables 1 and 2. [file 41563_2025_2138_MOESM1_ESM.pdf]

nature materials

Supplementary information https://doi.org/10.1038/s41563-025-02138-5

Microscopic crystallographic analysis of dislocations in molecular crystals

In the format provided by the authors and unedited

Supplementary Information Table of Contents

Contents

Contents	Page
**Supplementary Note 1**	2
**Supplementary Figure 1**	3
**Supplementary Figure 2-5**	6
**Supplementary Figure 6-7**	11
**Supplementary Table 1**	12
**Supplementary Note 2**	13
**Supplementary Figure 8-9**	17
**Supplementary Table 2**	20
**Supplementary Note 3**	21
**Supplementary Figures 10-28**	21
**Supplementary Note 4**	40
**References**	41

Supplementary Note 1: Bend contour method for Burgers vector analysis

Measurements of bend contour displacement and azimuthal angle

Bend contour displacements were measured as the distance along the dislocation core between two lines marking the central path of the bend contour, with bend contours typically appearing as approximately parallel lines on either side of the dislocation core. In cases where twisting of the bend contour at the dislocation line was present, the middle of the bend contour was determined away from dislocation core (two red dashed lines in Supplementary Figure 1b) to avoid overestimation of the displacement measurement by the short-range deflections of the bend contour ('twisting') caused by the dislocation strain field. Supplementary Figure 1c shows a challenging case where no gap in intensity appears, but an offset at the dislocation core was still observable. Moreover, this example was taken from the edge of a field of view with only part of the bend contour to the right of the dislocation core included in the dataset. As such, the bend contour to the right of the dislocation line was taken as parallel to the bend contour to the left (as for Supplementary Figure 1b), but with the centre estimated at the right-hand edge of the field of view. Such cases may introduce some loss of precision in the displacement measurements but did not preclude construction of a polar plot for analysis of the dislocation Burgers vector (see also Supplementary Figure 20).

In conducting the measurement, we note that the precision depends on accurately locating the centre of the bend contour for displacement measurements. For symmetric bend contour, the precision of determining the centre can be much better than the diffraction-limited probe resolution. The bend contours can be wide, but their symmetry allows for precise determination of the center. Going to higher-order diffraction vectors can help sharpen the bend contours (i.e. narrow their width) and generally results in better constraint of the center of the bend contours, even in the cases for asymmetric bend contours, though node patterns (such as those with higher order $\mathbf{g}_{hkl} \cdot \mathbf{B} = n$ conditions) may obscure the precise dislocation line. A combination of lower and higher order reflections can typically refine the dislocation line to precisions much greater than the width of the bend contour. Small-scale changes in a bend contour's path (tortuosity) near a dislocation core likewise presents challenges to localization and may need to be excluded from the analysis (as for bend contours that cross a dislocation at the edge of or outside the field of view).

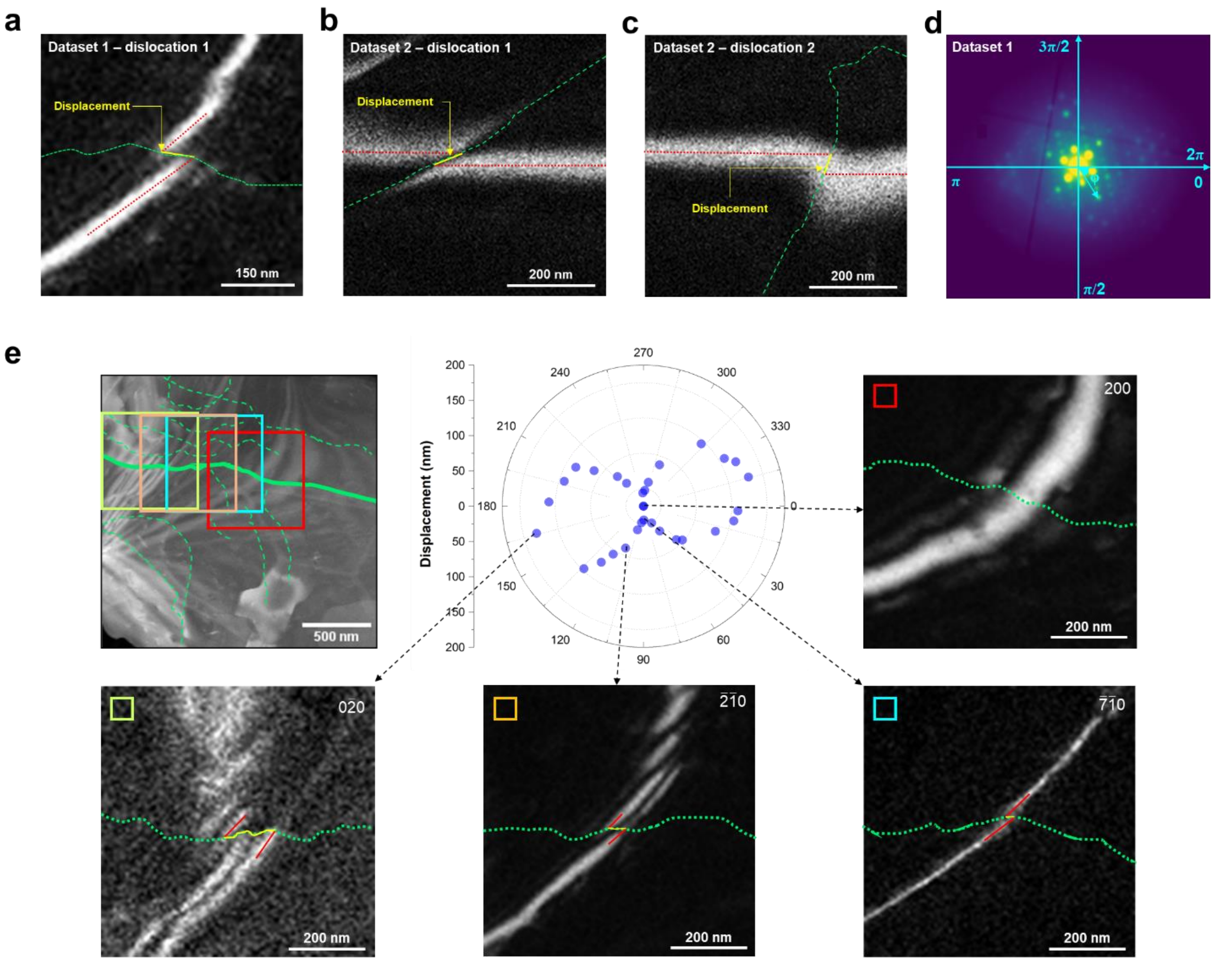

Supplementary Figure 1. Measurements of bend contour displacements. **a**, An example in a p-terphenyl film showing a symmetrical break of the bend contour along the dislocation line (Dataset 1 is also shown in Fig. 2). **b**, An example in an anthracene dataset showing a symmetrical break and twisting of the bend contour along the dislocation line (Dataset 2 is also shown in Figure 2, denoted as dislocation-1) **c**, An example in an anthracene dataset showing displacement of the bend contour direction on passing the dislocation line without a gap in the intensity profile (Dataset 2 as in **b**; this dislocation is denoted dislocation-2 and is also analysed in Supplementary Figure 20). **d**, Illustration of the definition of the azimuthal angle φ as the angle between a selected $\mathbf{g}_{hkl}$ vector and the horizontal axis of the diffraction pattern (shown using the Dataset 1 average pattern, also presented in Fig. 2). **e**, VDF images formed by placing virtual apertures at different $\mathbf{g}_{hkl}$ and cropped at marked areas (colour-coded boxes in ADF image) showing an increasing trend in the bend contour displacement when moving away from $\mathbf{g}_{200}$.

Geometric model of bend contour displacement

The bend contour approach relies on observing the tilt in planes at a dislocation core through (often) large displacements of a bend contour across the core (10 nm to more than 100 nm). The bend contours are typically at an angle to the dislocation line, and we can localise the dislocation line from the abrupt shift and termination of the bend contour, even when the bend contours are wide or overlapping. This approach differs from other dislocation imaging approaches like the weak-beam dark-field method where the sharpness of dark-field contrast depends on the diffraction order (for higher order reflections only planes that are highly tilted in close proximity to the core meet the Bragg condition and produce a bright signal in the weak beam image).

Based on the principle of plane tilting at the core, we introduce a geometric solution for the bend contour displacement on crossing dislocations in Supplementary Figure 2-5. Simple representation of the atomic displacement via $\mathbf{B}$ direction is shown for edge-type dislocation (Supplementary Figure 2) and screw-type dislocation (Supplementary Figure 5). From the viewing projection along the Cartesian z-axis for both dislocation types defined in the xy-plane, a trigonometric relationship can be derived between the Burgers vector $\mathbf{B}$ and the angle between $\mathbf{B}$ and the plane normal ($\mathbf{g}_{hkl}$):

$$x = \chi\mathbf{B}\cos^2(\varphi) \qquad \text{(S-1)}$$

with $\chi\mathbf{B}$ is a constant for any fixed set of displacements for a given dislocation. We note that this construction arises from the geometry defined by the lattice sites below (filled circles, Supplementary Figure 2) and above (unfilled circles, Supplementary Figure 2) the plane defined by the dislocation line **u** and the Burgers vector $\mathbf{B}$. These positions, in a defect free crystal, are in the same plane. In the system containing a dislocation the Burgers vector induces a displacement (perpendicular to the dislocation line for the edge dislocation shown). As such, the geometry arises from the displacement relative to the defect-free plane as projected onto the plane defined by $\mathbf{g}_{hkl} \cdot (\mathbf{B} \times \mathbf{u}) = 0$. As these geometric features are defined by the dislocation ($\mathbf{B}$ and **u**) and positions displaced relative to an original plane $\mathbf{g}_{hkl}$, the model does not rely on any assumptions about the angles between planes (Supplementary Figure 2e). As such the geometry is general for all crystal systems. For a perfect crystal with no defects presented, local bending of the sample, at the angles larger than the Bragg angle, defines a set of diffraction conditions for a particular set of planes where the planes are brought into or through the Bragg condition. This condition produces bend contours running across images of the film. Putting it another way, the bend contour is formed by the tilting of the lattice planes, due to local bending, to an angle θ_{hkl} along the $\mathbf{g}_{hkl}$ to intersect with the Ewald sphere at zero excitation error. The strain field around a dislocation core produces additional local tilt of surrounding planes as 'seen' by the $\mathbf{g}_{hkl}$. The additional tilts of planes at the core cause a shift in the bend contour at the dislocation line to match the new angle required to satisfy the Bragg condition exactly. Following the decomposition of the $\mathbf{g}_{hkl}$ into the vector parallel to $\mathbf{B}$ ($\mathbf{g}^{||\mathbf{B}}$) and the vector perpendicular to $\mathbf{B}$ ($\mathbf{g}^{\perp\mathbf{B}}$). It can be noticed that the component $\mathbf{g}^{||\mathbf{B}}$ changes under tilting due to the dislocation and the component $\mathbf{g}^{\perp\mathbf{B}}$ is unchanged. To have the same angle as where $\mathbf{g}_{hkl}$ intersects the Ewald sphere requires a change in $\mathbf{g}^{||\mathbf{B}}$ only which is then linked to the angle $\theta^{||\mathbf{B}}$, and is thus, linked to the parameter x. We can now calculate the $\theta^{||\mathbf{B}}$ for the specific hkl planes (Supplementary Figure 3) as:

$$\theta^{||\mathbf{B}}{}_{hkl} = \arctan\left(\frac{x}{d_\perp}\right) \qquad \text{(S-2)}$$

with $d_\perp$ is the d-spacing that allows for completing the triangle to translate the x parameter into an angle $\theta^{||\mathbf{B}}$. The vertical R line is taken in Supplementary Figure 4 as a reference angle for the exact Bragg condition (bend contour criterion) while the red line and blue line indicate the tilted planes

at either sides of the dislocation core. The shift from the vertical R line will be approximately the arc length Rθ for tilt angle θ for local tilting caused by dislocation is $\theta^{||\mathbf{B}}$. Hence, the total shift between the red and blue tilted planes at the dislocation core will be ~2R$\theta^{||\mathbf{B}}$. We can now construct the function for total shift/displacement of the bend contour on crossing the dislocation as:

$$f(\varphi) = 2R\theta^{||\mathbf{B}} = 2R \arctan\left(\frac{x}{d_{\perp}}\right) = 2R \arctan\left(\frac{\chi\mathbf{B}\cos^2(\varphi)}{d_{\perp}}\right) \quad \text{(S-3)}$$

The function f(φ) can be simplified to: $f(\varphi) = A\arctan(B\cos^2(\varphi - C))$ where A, B, and C are fitting coefficients. We note that in most applications without controlled (and therefore not necessarily uniform) curvature, the fitting coefficient A will represent an *effective* curvature. Provided the changes in the radius of curvature remain slowly varying across the field of view (a gentle bending condition), we expect the variations in R across the field of view to reduce the quality of the fit without precluding the extraction of the Burgers vector direction. Without control of the curvature through bespoke modifications to sample preparation or separate measurement of the curvature, we do not recommend quantifying the curvature from the fitting of A directly.

From equation S-3, it can be noticed that f(φ) will be 0 when φ is 90°, i.e. the $\mathbf{g}_{hkl}$ perpendicular to the Burgers vector ($\mathbf{g}_{hkl} \cdot \mathbf{B} = 0$), which is equivalent to when the Burgers vector lies parallel to the planes. It is in agreement with the definition for the invisibility criterion that states: the strain field around the dislocations causes local deviations or distortion of diffracting planes from their regular arrangement unless the Burgers vector of the dislocations lies parallel to the diffracting planes[1]. As such, the bend contours, associated to these planes, show no break on crossing the dislocation.

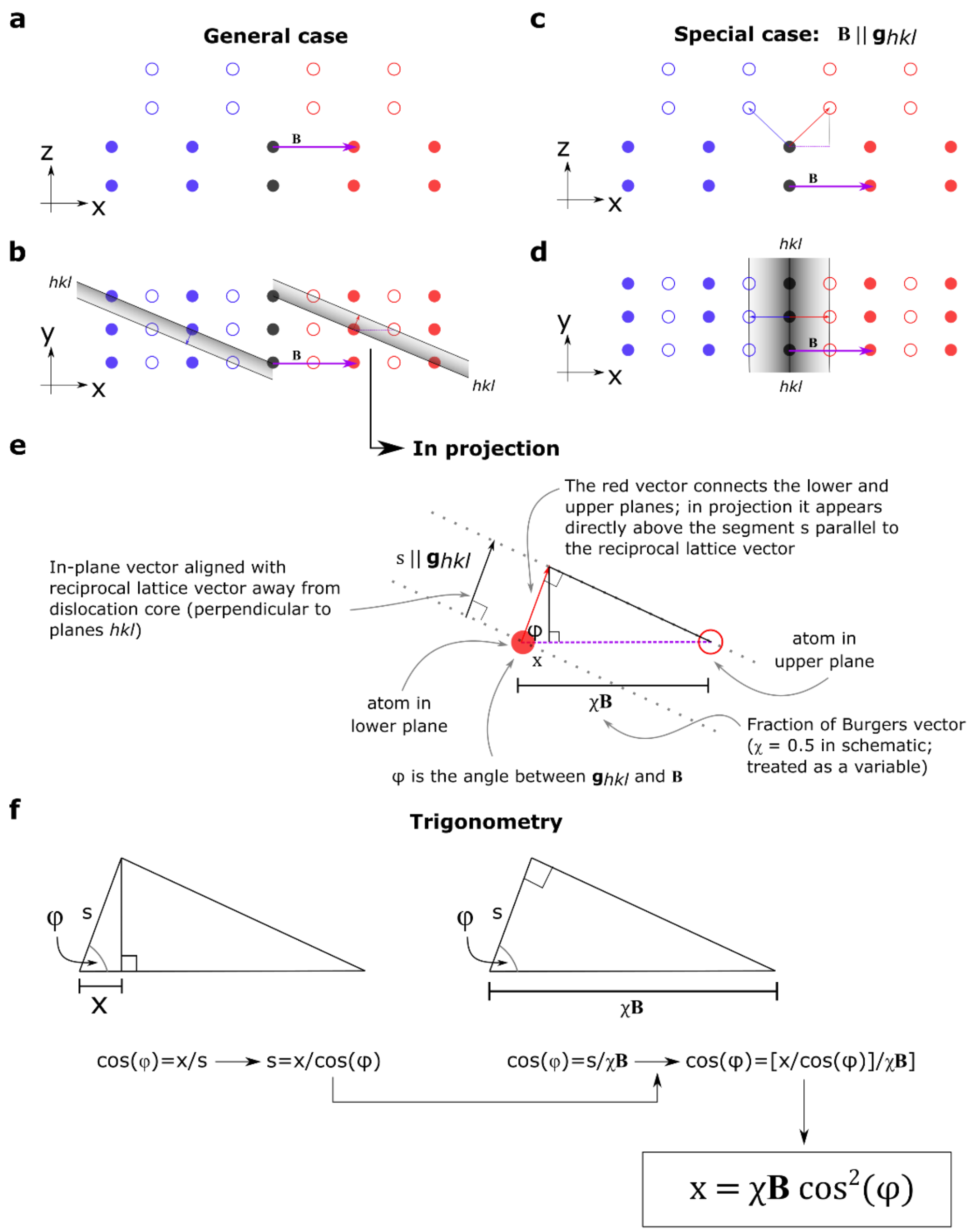

Supplementary Figure 2. Construction of a geometric model for lattice plane displacements due to dislocations. a, Side-on and **b,** plan view schematics of an edge dislocation. The purple arrow marks the Burgers vector **B.** Filled circles denote lattice sites below the dislocation core and unfilled circles denote lattice sites above the dislocation core. Red and blue colours mark lattice sites to the right and left of the dislocation line, respectively, with black circles marking the sites aligned with the dislocation line. Shaded planes in **b** indicate the tilting of a selected plane outlined by the circles below the dislocation line and the circles above the dislocation line (shifted relative to their defect-free positions). Red and blue arrows connect the lower and upper lattice sites (i.e. arrows lie in the shaded, tilted planes). A dashed purple line marks the projection of the Burgers vector. **c**, Side-on and **d**, plan view schematics of the special case where the selected planes are maximally tilted. This case is defined by the plane normal in the defect-free material and the coincident scattering vector $\mathbf{g}_{hkl}$ parallel to the Burgers vector. Dashed purple and grey lines in **c** decompose the red arrow connecting the lower to upper lattice sites into projections along the Burgers vector and perpendicular to the Burgers vector. **e**, Geometric relationships fin projection (i.e. the plan view perspective for an in-plane dislocation line) between the planes above and below the dislocation core. The Burgers vector and the scattering vector $\mathbf{g}_{hkl}$ define an in-plane angle φ. Decomposition of the trigonometric relationship establishes a partial displacement *x* along the Burgers vector direction (itself a fixed fraction of the total Burgers vector $\chi\mathbf{B}$). **f**, Trigonometric rearrangements to define the displacement *x* in terms of $\chi\mathbf{B}$ and φ.

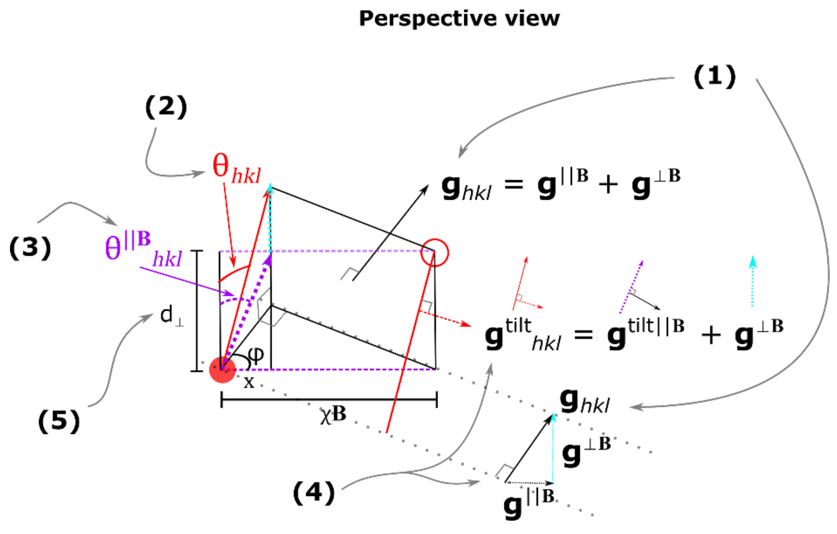

Step-wise commentary

(1) Away from the dislocation core, the reciprocal lattice vector (scattering vector) can be decomposed along (parallel to) and perpendicular to the Burgers vector (geometric decomposition defined by $\mathbf{B}$)

(2) At the dislocation core, the planes *hkl* are tilted by a total angle θ_{hkl}

(3) The tilt can be decomposed into components along the Burgers vector and perpendicular to the Burgers vector (geometric decomposition defined by $\mathbf{B}$)

(4) At the dislocation core, the reciprocal lattice vector (scattering vector) is tilted by the angle corresponding to the tilt of the planes hkl and can be decomposed in the same way; only the component parallel to the Burgers vector changes relative to the geometry away from the dislocation core

(5) A perpendicular distance (geometric variable, i.e. not crystallographic) can be used to link *x* and the isolated component tilt parallel to the Burgers vector (tilt angle into the plane parallel to the Burgers vector). Equivalently, the tangent of the angle exists even where it is an unkown variable of the system:

$$\tan(\theta^{||\mathbf{B}}_{hkl}) = x/d_{\perp} \rightarrow \theta^{||\mathbf{B}}_{hkl} = \arctan(x/d_{\perp})$$

Supplementary Figure 3. Geometric model for bend contour displacements arising from dislocations in the bending crystals. The perspective view drawing of the geometry corresponds to the plan view drawing in Supplementary Figure 2e. Stepwise commentary on the right provides a guide to the geometric link between the selected tilted plane and the tilt in the associated plane normal and scattering vector $\mathbf{g}^{tilt}_{hkl}$.

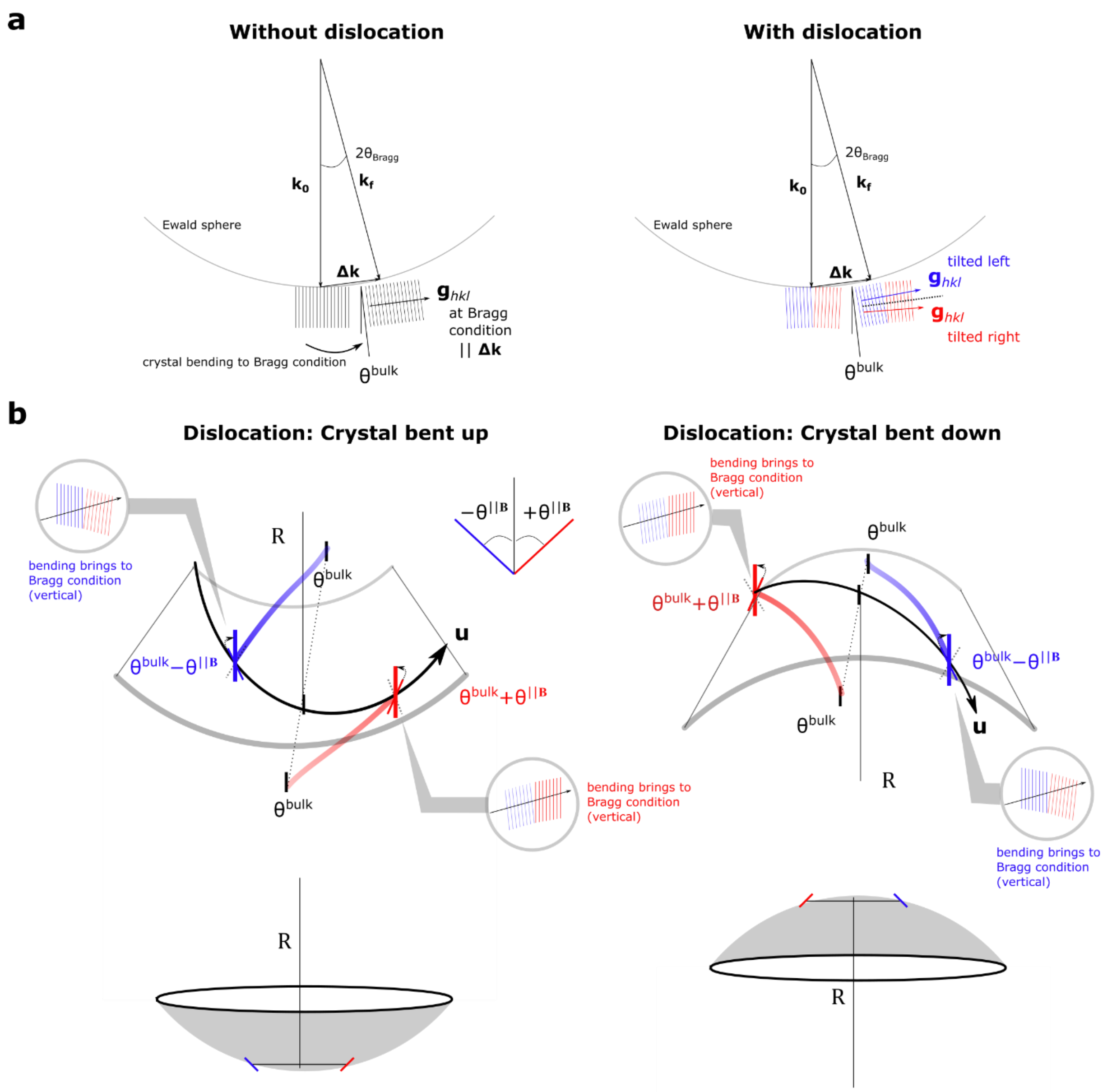

Supplementary Figure 4. Schematic of the bend contour displacement on crossing a dislocation line. a, The Ewald sphere defines the Bragg condition where the change (**Δk**) from the incident wavector $\mathbf{k_0}$ to the final wavector $\mathbf{k_f}$ is equal to the diffraction vector $\mathbf{g}_{hkl}$. A bend contour arises when the crystal bending brings the corresponding planes (*hkl*) to the necessary Bragg condition, requiring a tilt to θ_{bulk} in the absence of a dislocation. In the presence of a dislocation (illustrated as an edge dislocation as in Supplementary Figure 2-3), the planes and diffraction vectors on either side of the dislocation line are tilted relative to the planes away from the dislocation core. As drawn, the blue planes (left of the dislocation line) are tilted to the left and the red planes (right of the dislocation line) are tilted to the right. **b**, Illustrations of how the tilted planes at the dislocation core abruptly change the location of the Bragg condition in a bent crystal resulting in a displacement in the bend contour. The illustrations show the case of a crystal bent 'up' or 'down'. The sample is assumed to be described by a local radius of curvature R. The vertical direction is selected as a reference to the Bragg condition in the crystal without a dislocation (θ_{bulk}) for a set of planes (*hkl*) corresponding to diffraction vector $\mathbf{g}_{hkl}$. The incident electron beam direction is not shown in **b.** Tilting to the right is considered as an additional positive tilt $+\theta^{||\mathbf{B}}$ and tilting to the left is considered as an additional negative tilt $-\theta^{||\mathbf{B}}$**.** Planes tilted to positive (red) or negative (blue) angles at the dislocation core (line **u**) require compensating tilts from crystal bending to match the Bragg condition. These tilts displace the Bragg condition on the sample in opposite and equal parts (assuming locally symmetric curvature). This displacement is considered as an arc length (for sphere of radius R; a good approximation for small curvatures, taken as the projected arc length): $2R\theta^{||\mathbf{B}}$. A large value of R corresponds to a flat (low curvature) surface, resulting in large displacements to compensate tilting of planes in opposite directions on either side of the dislocation core. Geometric features are exaggerated for visual effect in the schematic.

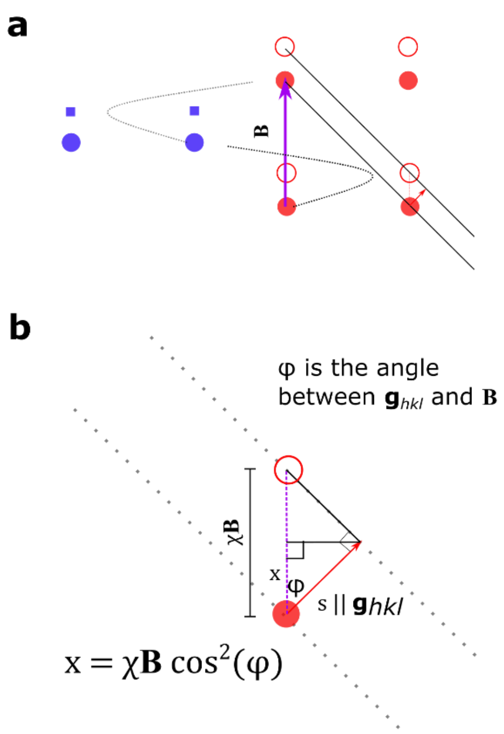

Supplementary Figure 5. Illustration of the geometric model for a screw dislocation. a, A view of a screw dislocation in projection with the dislocation line in the plane of the page. The purple arrow marks the Burgers vector $\mathbf{B}$**.** Filled red circles denote lower lattice sites (in the plane of the page) and unfilled red circles denote raise lattice sites (out of plane, above the page) around the helical lattice distortion at the dislocation core. Filled blue circles denote lower lattice sites in the plane of the page and filled blue squares denote raised lattice sites (out of plane, below the page) around the helical distortion at the dislocation core. Red and blue colours mark lattice sites to the right and left of the dislocation line, respectively. A fine dashed line marks the helical path of successive lattice sites. A red arrow connects the lower and upper lattice sites on the right. **b,** Geometric relationships in projection (i.e. an in-plane dislocation line) between the planes marked in **a**. A dashed purple line marks the projection of the Burgers vector. The Burgers vector and the scattering vector $\mathbf{g}_{hkl}$ define an in-plane angle φ. Decomposition of the trigonometric relationship establishes a partial displacement *x* along the Burgers vector direction (itself a fixed fraction of the total Burgers vector $\chi\mathbf{B}$). The same trigonemtric relationship is established as in Supplementary Figure 2 for an edge dislocation.

Burgers vector determination

The Burgers vector $\mathbf{B}$ is determined from the invisibility criterion condition $\mathbf{g}_{hkl} \cdot \mathbf{B} = 0$ where $\mathbf{g}_{hkl}$ at this condition can be found directly from the analysis of VDF images or can be estimated by the curve fitting using function f(φ). For extended, in-plane dislocations ($\mathbf{g}_{hkl} \cdot (\mathbf{B} \times \mathbf{u}) = 0$), the Burgers vector direction can be recovered unambiguously. In this case, the Burgers vectors are also in-plane and perpendicular to the beam direction $\mathbf{z}$, i.e. $\mathbf{B} \times \mathbf{u} \parallel \mathbf{z}$. Thus, we can equivalently define the condition $\mathbf{B} \cdot \mathbf{z} = 0$ with $\mathbf{z}$ taken as the viewing direction (zone axis direction). For $\mathbf{B}$ = [u_1 v_1 w_1], $\boldsymbol{z}$ = [u_2 v_2 w_2], and $\mathbf{g}$ = (*hkl*), we obtain set of equations:

$$\begin{cases} hu_1 + kv_1 + lw_1 = 0 \\ (u_1\mathbf{a}+v_1\mathbf{b}+w_1\mathbf{c}).(u_2\mathbf{a}+v_2\mathbf{b}+w_2\mathbf{c})=0 \end{cases} \quad \text{(S-4)}$$

with **a, b,** and **c** are the unit cell translations in real space. Since $\mathbf{z}$ and $\mathbf{B}$ are represented in the lattice vector basis, u_1, v_1, w_1, u_2, v_2, and w_2 are all integers. Expanding the second equation in S-4, we have:

$$u_1.\,u_2.|\mathbf{a}|^2 + (u_1v_2 + v_1u_2).|\mathbf{a}|.|\mathbf{b}|.\cos(\gamma) + (u_1w_2 + w_1u_2).|\mathbf{a}|.|\mathbf{c}|.\cos(\beta)$$
$$+ v_1.v_2.|\mathbf{b}|^2 + (v_1w_2 + w_1v_2).|\mathbf{b}|.|\mathbf{c}|.\cos(\alpha) + w_1.w_2.|\mathbf{c}|^2 = 0 \quad \text{(S-5)}$$

We can now notice that equation S-5 can be simplified to $u_1u_2 + v_1v_2 + w_1w_2 = 0$ if $\alpha = \beta = \gamma = 90^o$ and $|\mathbf{a}| = |\mathbf{b}| = |\mathbf{c}|$, i.e. a cubic crystal. However, the studied molecular crystals in this study have non-cubic structure. As a result, equation S-5 must be used when calculating the Burgers vector $\mathbf{B}$. For given crystal structures, unit cell parameters, and zone axes from the studied materials, sets of equations to calculate $\mathbf{B}$ can be deduced as laid out below:

- For p-terphenyl (monoclinic) and *n*-hentriacontane (orthorhombic) viewed along $\mathbf{z}$ = [001], S-5 becomes:

$$\begin{cases} hu_1 + kv_1 + lw_1 = 0 \\ u_1.|\mathbf{a}|.\cos(\beta) + w_1.|\mathbf{c}| = 0 \end{cases} \quad \text{(S-6)}$$

- For anthracene (monoclinic) viewed along $\mathbf{z}$ = [101], S-5 becomes:

$$\begin{cases} hu_1 + kv_1 + lw_1 = 0 \\ u_1.|\mathbf{a}|^2 + (u_1 + w_1).|\mathbf{a}|.|\mathbf{c}|.\cos(\beta) + w_1.|\mathbf{c}|^2 = 0 \end{cases} \quad \text{(S-7)}$$

- For theophylline form II (orthorhombic) viewed along $\mathbf{z}$ = [141], S-5 becomes:

$$\begin{cases} hu_1 + kv_1 + lw_1 = 0 \\ u_1.|\mathbf{a}|^2 + 4.v_1.|\mathbf{b}|^2 + w_1.|\mathbf{c}|^2 = 0 \end{cases} \quad \text{(S-8)}$$

- For theophylline form IIIb (monoclinic) viewed along $\mathbf{z}$ = [211], S-5 becomes:

$$\begin{cases} hu_1 + kv_1 + lw_1 = 0 \\ 2u_1.|\mathbf{a}|^2 + (u_1 + 2w_1).|\mathbf{a}|.|\mathbf{c}|.\cos(\beta) + v_1.|\mathbf{b}|^2 + w_1.|\mathbf{c}|^2 = 0 \end{cases} \quad \text{(S-9)}$$

- For theophylline form M (monoclinic) viewed along $\mathbf{z}$ = $[\bar{2}13]$, S-5 becomes:

$$\begin{cases} hu_1 + kv_1 + lw_1 = 0 \\ -2u_1.|\mathbf{a}|^2 + (3u_1 - 2w_1).|\mathbf{a}|.|\mathbf{c}|.\cos(\beta) + v_1.|\mathbf{b}|^2 + 3w_1.|\mathbf{c}|^2 = 0 \end{cases} \quad \text{(S-10)}$$

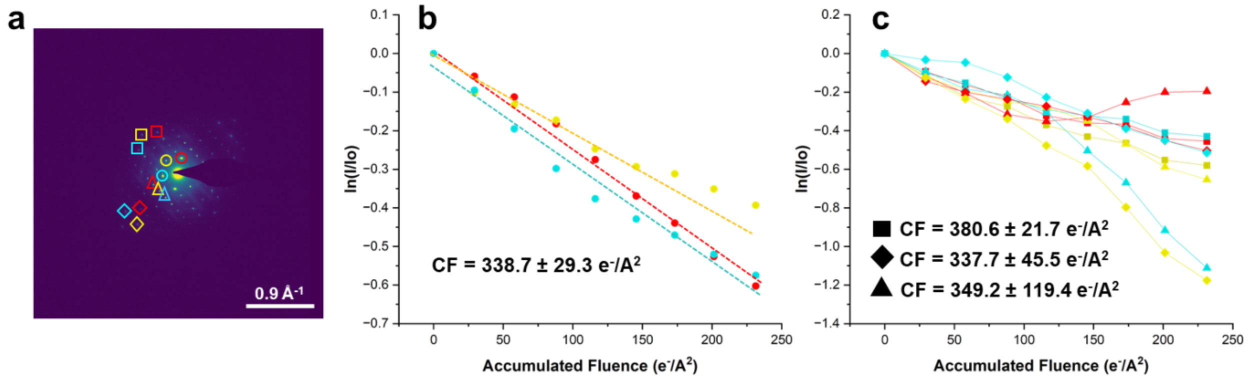

Supplementary Figure 6. Loss of Bragg diffraction intensity with cumulative electron fluence (beam-damage series) for a p-terphenyl thin film viewed along [001]. a, Selected Area Electron Diffraction Pattern (SAED) taken at the beginning of the experiment (t_o) showing the marked diffraction spots for CF analysis. **b**, Decay profile of the lattice planes having *d*-spacing > 4 Å. **c**, Decay profile of the lattice planes having *d*-spacing < 4 Å. The square-marked denote the diffraction spots corresponding to lattice planes with *d*-spacings 1.3-1.6 Å. The diamond-marked spots denote the diffraction spots corresponding to lattice planes with *d*-spacings 1.1-1.4 Å. The triangle-marked spots denote the diffraction spots corresponding to the lattice planes with *d*-spacings 2.6-2.9 Å. The decay profiles show an approximately log-linear response with adjusted determination coefficient $R^2 > 0.9$. The CF is extracted from the gradient of the linear fit (see also Supplementary Note 4).

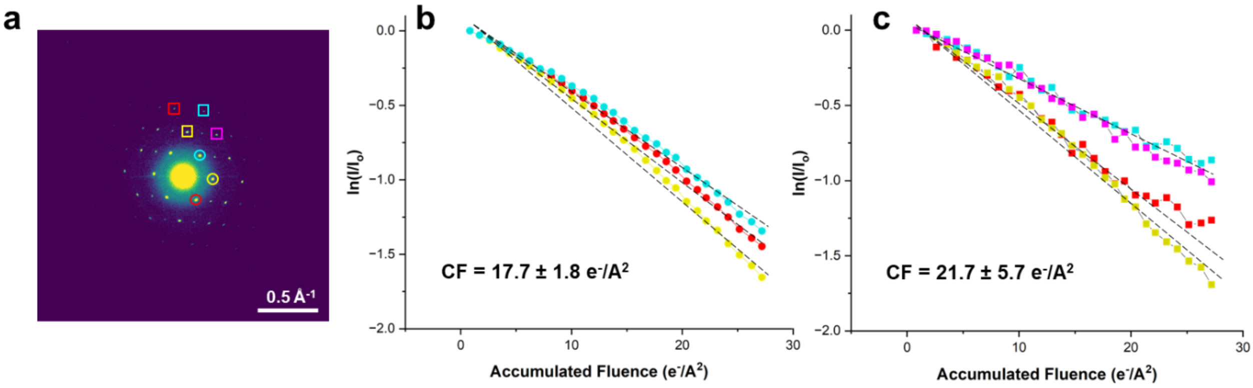

Supplementary Figure 7. Loss of Bragg diffraction intensity with cumulative electron fluence (beam-damage series) for an anthracene thin film viewed along [101]. a, Selected Area Electron Diffraction Pattern (SAED) taken at the beginning of the experiment (t_o) showing the marked diffraction spots for CF analysis. **b**, Decay profile of the lattice planes having *d*-spacing > 4Å. **c**, Decay profile of the lattice planes having *d*-spacing < 4Å. The decay profiles showed an approximately linear response with an adjusted determination coefficient (R^2) > 0.9. The CF is extracted from the gradient of the linear fit (see Supplementary Note 4).

Supplementary Table 1. Estimated in-plane dislocation densities for molecular crystal samples. The dislocation density is determined from measurement of the total length of all observed in-plane dislocations per unit volume of the crystal. The volume was estimated from the area of the field of view and an estimated sample thickness *T*, taken as a range from 10-100 nm. This estimate was consistent with thickness contrast from amorphous carbon support films (lacey carbon). Thickness likely varies within and between samples, and we report the dislocation densities here as order of magnitude estimates.

**Figure**	**Length (nm)**	**Area (nm^2)**	***T* (nm)**	**Volume (nm^3)**	**Dis. Density (cm^{-2}) ($x10^{10}$)**	**Sample**
Fig. 2b	12777	4.0×10^6	10-100	$4.0 x10^7$-$4.0 x10^8$	0.3-3.2	p-terphenyl
Fig. 2e	2594	10^6	10-100	10^7-10^8	0.3-2.6	anthracene
Fig. 3b	1040	2.0×10^6	10-100	$2.0\ x10^7$-$2.0 x10^8$	0.1-0.5	theophylline
Fig. 3f	3924	10^6	10-100	10^7-10^8	0.4-3.8	*n*-hentriacontane (wax)
Supp. Fig. 11a	1040	10^6	10-100	10^7-10^8	0.1-1.0	p-terphenyl
Supp. Fig. 12a	1640	10^6	10-100	10^7-10^8	0.2-1.7	p-terphenyl
Supp. Fig. 14	10821	7.0×10^6	10-100	$7.2 x10^7$-$7.2 x10^8$	0.2-1.5	p-terphenyl
Supp. Fig. 21a	4221	4.0×10^6	10-100	$4.0 x10^7$-$4.0 x10^8$	0.1-1.1	anthracene
Supp. Fig. 22a	10560	1.5×10^6	10-100	$1.5 x10^7$-$1.5 x10^8$	0.7-6.9	theophylline
Supp. Fig. 23a	978	1.3×10^6	10-100	$1.3 x10^7$-$1.3 x10^8$	0.1-0.7	theophylline
Supp. Fig. 25a	13889	2.2×10^6	10-100	$2.2 x10^7$-$2.2 x10^8$	0.6-6.3	1-triacontanol (wax)
Supp. Fig. 26a	1944	3.8×10^6	10-100	$3.8 x10^7$-$3.8 x10^8$	0.1-0.5	p-terphenyl
Supp. Fig. 27a	2040	0.5×10^6	10-100	$0.5 x10^7$-$0.5 x10^8$	0.4-4.5	anthracene

Supplementary Note 2: Goodness-of-fit evaluation for polar function fitting

We report the fitting function (equation 1) $f(\varphi) = A\arctan(B\cos^2(\varphi - C))$ arising from the geometric model for the bend contour displacement on crossing the dislocations (Supplementary Figures 2-5) to estimate the Burgers vector direction from experimental displacement measurements. We have evaluated the goodness-of-fit of the proposed model using goodness-of-fit statistics and together with inspection of the residuals, and evaluation of uncertainties in fitting parameter estimation. For goodness-of-fit statistics, the coefficient of determination R^2 and the chi-square (χ^2) test statistic are used. Meanwhile, visualization of the fitting on the measured data and residual analysis are used as graphical methods to evaluate the fit.

R^2 statistic

The R^2 statistic seeks to summarise how close the fitted curve passes through the recorded measurements, most commonly used in linear regression analysis. R^2 can be defined from the sum of squared residuals (SS_{res}) and the total sum of squares (SS_{tot}):

$$R^2 = 1 - \frac{SS_{res}}{SS_{tot}} = 1 - \frac{\sum_i (y_i - f_i)^2}{\sum_i (y_i - \bar{y})^2} \quad \text{(S-11)}$$

with y_i is the measured data, f_i is the predicted data from the fitting model, and $\bar{y}$ is the mean of the measured data. Where the predicted values determined from the fitting model exactly match the measured values, the $SS_{res} = 0$ and $R^2 = 1$. We report this statistic as an indicator of the match between experiment and the fitted curve, while noting its limitations in distinguishing distributed errors arising from experimental uncertainty and systematic errors in a poor model correspondence to the recorded measurements.

χ^2 statistic

The χ^2 statistic offers a route to evaluate a fitted curve in the context of variance in experimental measurements. The simplest χ^2 can be calculated as follows:

$$\chi^2 = \sum_i \frac{(y_i - f(x_i))^2}{\sigma_i^2} \quad \text{(S-12)}$$

where y_i is again the measured data, $f(x)$ is the fitting model, x_i is the independent variable, and σ_i is the uncertainties/standard variation. For the purposes of the evaluation of curve fitting, the chi-square per degrees of freedom or reduced chi-square is useful as it takes into consideration the number of data points (measurements) and number of parameters describing the fitted function. The reduced chi-square can be calculated by dividing the chi-square to the number of degrees of freedom ν:

$$\chi^2_{red} = {\chi^2}/{\nu} \quad \text{(S-13)}$$

where $\nu = N - n_p$ for N data points and n_p parameters in the fitting model. A model (curve) that fits the data within the variance inherent in the data will exhibit a χ^2_{red} near 1. A χ^2_{red} much greater than 1 will indicate a poor agreement between the model and the measurements, and a χ^2_{red} less

than 1 will indicate the model fits more closely to the data than expected for the inherent variation in the measurements.

To be able to calculate the χ^2, and thus χ^2_{red}, it is essential to estimate the variance σ_i^2 in the measured data, i.e. the variance in the measured bend contour displacements. The variance of the bend contour displacements likely differ for different bend contours (different $\mathbf{g}_{hkl}$) and also for different sample areas, given by the variation in the width of bend contours due to the sample curvature as well as complex diffraction contrast in some areas of the samples. We estimated the variance based on an assumption that symmetrical bend contours (symmetry-equivalent $\mathbf{g}_{hkl}$) are expected in the model to have a single value, i.e. a mean bend contour displacement for symmetrical bend contours). A minimum of 3 $\mathbf{g}_{hkl}$ and corresponding bend contours would in principle allow an estimate of the variance for a particular $\mathbf{g}_{hkl}$. However, for the data presented here, few of the datasets show 3 or 4 measured for symmetry-equivalent $\mathbf{g}_{hkl}$ while the majority of datasets show only one or no available sets of symmetrical bend contour displacements. As a result, we evaluated the uncertainties across all datasets with the aim of estimating a global variance σ^2_{global} for the general measurement of bend contour displacement in SED data. The variance for each symmetrical $\mathbf{g}_{hkl}$ follows:

$$\sigma^2 = \frac{1}{N}\sum_{i=1}^{N}(y_i - \bar{y})^2 \tag{S-14}$$

and the global variance is the average value of all the variances estimated from available symmetrical $\mathbf{g}_{hkl}$ across the datasets. Using this approach, we estimated the global variance σ^2_{global} to be 71.2. This global variance corresponds to a global standard deviation σ_{global} of 8.4. In turn, we used this global variance estimate for calculating χ^2_{red}. We recognised that this estimate is imperfect due to differences in scatter of measurements for different bend contours within a SED dataset and between SED datasets, but we believe the χ^2_{red} nevertheless provides a useful summary statistic to evaluate the overall fitting approach and to compare fits with different number of parameters where necessary due to poorly constrained fitting parameters (high uncertainties in fitting parameters).

Evaluation of uncertainties in fitting parameters

The uncertainties in the fitting parameters in the simplified function f(φ) (equation 1) were calculated from the diagonals of the estimated covariance matrix of these parameters after fitting the data with the curve_fit function. The standard errors on the parameters were calculated by taking the square root of the diagonals of the covariance matrix. We also calculated the confidence interval for the fitting parameters using lmfit() function to determine how tightly the determined values of the fitting parameters are after fitting. In addition, a two-dimensional (2D) plot of the confidence region for each pair of fitting parameters was used to evaluate the dependence between the fitting parameters. For some datasets, fitting to equation 1 resulted in estimated standard errors for the fitting parameters larger than the fitting parameter magnitudes, suggesting

these parameters were not well-constrained in the fitting to equation 1. We note that phenomenologically, there may be inherent ambiguity in constraining the parameters given limited data or where the available data can be fitted well with a range of compensating parameter settings. This off-setting property is particularly possible for the parameters *A* and *B* in equation 1 which both contribute to the scaling of the 'lobes' in the polar plots. In these cases, we have accordingly reduced the number of fitting parameters to avoid physically unreasonable fitting results.

In summary, our fitting evaluation procedure followed the workflow outlined below:

i. Carry out an initial fit to equation 1 $f(\varphi) = A\,\arctan(B\cos^2(\varphi - C))$

ii. Evaluate the suitability of equation 1 for the dataset:

   a. Evaluate the standard errors in the fitting parameters from the estimated covariance matrix.

   b. Evaluate confidence intervals and contour plot using Lmfit-1.2.0[2], a Python package for non-Linear least-squares minimization and curve-fitting.

   c. Analyse the residuals: A good model fit will exhibit residuals distributed symmetrically about zero. A poor model fit may exhibit multi-modal residual distributions or may exhibit a distribution centred away from zero.

   d. Calculate summary statistics (R^2, χ^2_{red})

iii. If equation 1 fitting is unsuitable, reduce the number of fitting parameters by adopting instead the fitting function (small-angle approximation to equation 1):

$$f(\varphi) = AB\cos^2(\varphi - C) = A_1\cos^2(\varphi - C) \qquad \text{(S-15)}$$

iv. Evaluate the fit to equation S-15, including standard errors in parameters, Lmfit confidence intervals and contour plots, residuals, and summary statistics (R^2, χ^2_{red}).

Illustrative examples

We illustrate this workflow in two example datasets: One that exhibits the properties of a well-constrained fit to equation 1 and a second that exhibits poorly constrained fitting to equation 1 and required fitting to equation S-15 instead.

The dataset depicted in Fig. 2c for p-terphenyl offers an example of a well-constrained fit to equation 1. Fitting bend contour displacements to equation 1 $f(\varphi) = A\,\arctan(B\cos^2(\varphi - C))$ gave the parameters *A* = 144 ± 10 (nm); *B* = 1.6 ± 0.2 (ratio); *C* = 2.94 ± 0.01 (rad). The confidence interval calculated for these fitting parameters show:

	95.45%	68.27%	_BEST_	68.27%	95.45%
A (nm):	-18	-10	144	+12	+28
B (ratio):	-0.4	-0.2	1.6	+0.2	+0.4
C (rad):	-0.03	-0.01	2.94	+0.01	+0.03

In this case, the standard error calculated from the estimated covariance matrix closely resemble a 1σ range in the confidence intervals (68.3%). For the *B* and *C* parameters, the uncertainties are approximately linear when going from 1σ (68.3% confidence) to 2σ (95.0% confidence), and these values are fairly symmetric around the best fit values for fitting parameter B and C. There is an asymmetry distribution in the uncertainties of the fitting parameter A. The contour plots of confidence region, i.e. the maps of probability, for pairs of fitting parameters are shown in Supplementary Figure 8a to visualize the distribution of uncertainty and the correlations between the fitting parameters. These contour plots exhibit isotropic distributions for *A/C* and *B/C*, but a highly elliptical distribution for the *A/B* parameters, further elaborating the origin of the asymmetric confidence interval. Nevertheless, there remains a well-defined optimal fitting region for the *A* and *B* parameters.

The histogram of the residuals for fitting Fig. 2c with equation 1 is also plotted and compared to the residuals fitting with equation S-15 to further probe the relative goodness-of-fit for the two models (Supplementary Figure 8b). The residuals plots show a distribution centred at zero for equation 1, and show a bimodal, split distribution at negative and positive error for fitting equation S-15. The summary statistics (R^2, χ^2_{red}) likewise confirm these conclusions, with values (0.97, 0.96) for fitting to equation 1 and (0.93, 2.35) for fitting to equation S-15.

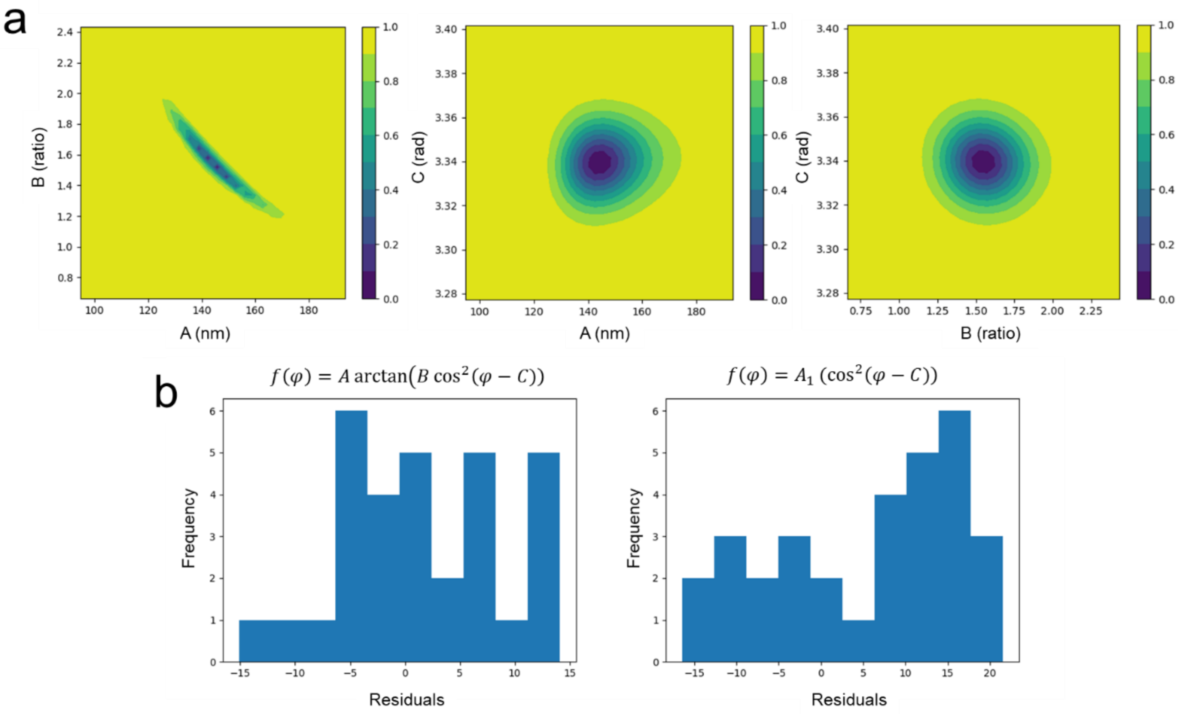

Supplementary Figure 8. Evaluation of fitting procedure applied to measurements in Fig. 2c. a, Contour plots of confidence region for each pair of fitting parameters showing the distribution of the uncertainties and the correlations between the fitting parameters: (left) correlation between A and B parameters, (middle) correlation between A and C parameters, and (right) correlation between B and C parameters. **b**, Histograms showing the distribution of the residuals for the two models used for the fitting.

Fig. 2g (anthracene) presents an example of a fit poorly constrained for fitting to equation 1 which required additional steps to finalise the fitting procedure. An initial fit to equation 1 gave the fitting parameters A = 166800 ± 2e+10 (nm); B = 0.0 ± 265.6 (ratio); and C = 0.69 ± 0.03 (rad). The confidence intervals calculated for these fitting parameters were determined as:

	95.45%	68.27%	_BEST_	68.27%	95.45%
A (nm):	-166568	-166360	166800	+inf	+inf
B (ratio):	-inf	-inf	0.0	+inf	+inf
C (rad):	-0.06	-0.03	0.69	+0.03	+0.06

These fitting results exhibited standard errors much larger than the A and B fitting parameter magnitudes. The fitting parameter C is well constrained, likely because this parameter defines the orientation of the 'lobes' in the polar plot. This orientation parameter appears to be well-separated from the scaling parameters A and B. The calculated confidence intervals for the A and B parameters are very asymmetric and approach negative and positive infinity (-inf and +inf). In other words, there is insufficient data to unambiguously determine the fitting parameters A and B in the model. Moreover, in the geometric model, the parameter A is associated with the local radius of curvature, which may not always be constant due to uncontrolled, inhomogeneous bending of the

sample. The sample bending could be dynamic during beam exposure, with local curvature changing over the time of the scan or successive images. The non-constant nature of the local curvature can create errors in fitting A and B. Effectively, many sets of *A* and *B* parameters will generate curves that fit the data equally well. The contour plots of confidence region (Supplementary Figure 9a) show no specific distribution for *A* and *B* which highlights that they are not well constrained.

In order to evaluate the effect of the initial guess on these results, we refined the fitting process by fitting fewer parameters. First, we fixed the *B* parameter and allowed *A* and *C* to vary in the fit and then used these values as initial guess values to fit all three parameters. Following the first step by fixing B = 0.5, we obtained the parameters *A* = 1005 ± 54 (nm) and C = 0.69 ± 0.02 (rad). These fitting results show reasonable estimates of the standard errors for *A* and *C*. After the second, three-parameter fit, the parameters became *A* = 169205 ± 2e+10 (nm); *B* = 0.0 ± 269.3; and *C* = 0.69 ± 0.03. These fitting results illustrate the instability in the fit, persistent for stepwise parameter fitting.

Consequently, we turned to a reduction in the number of parameter by simplification of the model. Parameters *A* and *B* together set the length and width of the 'lobes' in the polar plot. For small B or small values of $\cos^2(\varphi - C)$ the curve (equation 1) approaches the small-angle approximation: $f(\varphi) = A_1 \cos^2(\varphi - C)$ (equation S-15) which combines *A* and *B* into a single parameter A_1. Notably, $\cos^2(\varphi - C)$ will be small near $\mathbf{g}_{hkl} \cdot \mathbf{B} = 0$, providing suitable justification for fitting equation S-15 when measurements are only available near the zero-displacement condition for visible bend contours. More generally, incomplete sampling of the width and length of the 'lobes' by experimental measurements will offer poor constraints on *A* and *B*, and its replacement with a robust 2-parameter fit to equation S-15 retains the targeted form for identifying the $\mathbf{g}_{hkl} \cdot \mathbf{B} = 0$ as defined by $\cos^2(\varphi - C)$.

In Fig. 2g, by fitting equation S-15 we obtained A_1 = 493 ± 27 and *C* = 0.69 ± 0.02. The calculated confidence intervals were determined as:

	95.4%	68.3%	_BEST_	68.3%	95.5%
A_1 (nm):	-62	-28	493	+28	+62
C (rad):	-0.06	-0.03	0.69	+0.02	+0.05

The standard errors on the parameters are much smaller than the parameter values in this case, suggesting well-constrained parameter fits. The calculated confidence intervals also show approximately linear changes from 1σ (68.3% confidence) to 2σ (95.5% confidence), and these values exhibit symmetry around the best fit values for A_1 and *C*. The contour plots of confidence region A_1/C (Supplementary Figure 9b) exhibits an approximately isotropic distribution with no features indicating substantial correlation between A_1 and *C*. The residuals plots obtained for both fitting models are indistinguishable (Supplementary Figure 9c), highlighting the correspondence

between equation 1 and its small-angle approximation (equation S-15) for the available measurements in this example. The summary statistics (R^2, χ^2_{red}) were (0.92, 13.55) for fitting to equation 1 and (0.93, 12.04) for fitting to equation S-15, indicating similar summary statistics for both models albeit slightly improved for fitting to equation S-15. The χ^2_{red} in this final fitting (equation S-15) is notably greater than 1, indicating the residuals exceed the expected variance estimated by σ^2_{global}. The global variance may not correspond to the variance in the bend contour displacements in this dataset or across the available $\mathbf{g}_{hkl}$ for this dataset. Reviewed together, the equation S-15 fit offers a best fit with constrained parameters and unambiguously determines the orientation of the 'lobes' and therefore the $\mathbf{g}_{hkl} \cdot \mathbf{B} = 0$ condition. We note that across all datasets, the *C* parameter captures the key information for estimating the Burgers vector as it fits the orientation (azimuthal angle) of the 'lobes' of the polar plot.

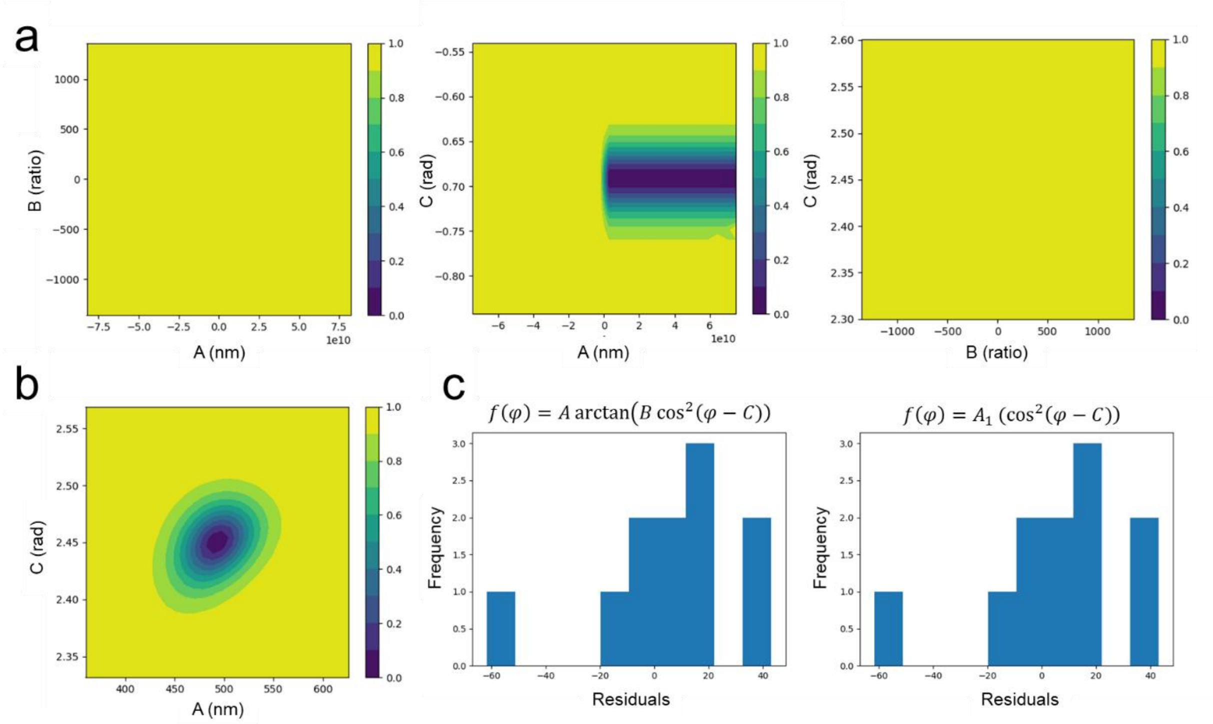

Supplementary Figure 9. Evaluation of fitting procedure applied to measurements in Fig. 2g. a, Contour plots of confidence region for each pair of fitting parameters using $f(\varphi) = A\arctan(B\cos^2(\varphi - C))$ as a fitting model: (left) correlation between A and B parameters, (middle) correlation between A and C parameters, and (right) correlation between B and C parameters. **b**, Contour plots of confidence region for A and C parameters using $f(\varphi) = A_1\,cos^2(\varphi - C)$ as a fitting model. **c**, Histograms showing the distribution of the residuals for two models used for the fitting.

Supplementary Table 2. Summary of fitting coefficients. The fitting coefficients are given with uncertainties reported as standard errors determined from the diagonals of the estimated covariance matrix. The coefficient of determination R^2, the number of data points *N*, degrees of freedom ν and χ^2_{red} are also reported. Rows containing fitting results for the parameter *B* report fitting to equation 1 (*A, B, C* parameters). Rows containing no *B* parameter fit show fitting to equation S-15 (A_1, *C* parameters). Rows containing a further *D* parameter used an additional form adapted for cases with two unresolved dislocation contributions (see Supplementary Figures 26-27).

Figure	***A*** **or** A_1 **(nm)**	***B*** **(ratio)**	***C*** **(rad)**	***D*** **(nm)**	R^2	***N***	**ν**	χ^2_{red}
Fig. 2c	144 ± 9.9	1.6 ± 0.2	2.94 ± 0.01	-	0.97	31	28	0.96
Fig. 2g	493 ± 26.6	-	0.69 ± 0.02	-	0.93	11	9	12.0
Fig. 3d	115 ± 7.8	2.6 ± 0.5	2.49 ± 0.03	-	0.98	14	11	0.81
Fig. 3h	144 ± 5.6	-	1.95 ± 0.02	-	0.97	21	19	0.94
Supp. Fig. 10-2	148 ± 2.2	-	2.96 ± 0.01	-	0.98	38	36	0.81
Supp. Fig. 10-3	111 ± 20.4	1.3 ± 0.4	2.91 ± 0.02	-	0.94	23	20	1.15
Supp. Fig. 11b-1	67 ± 9.3	-	2.74 ± 0.05	-	0.85	15	13	0.39
Supp. Fig.11b-2	115 ± 36.7	1.5 ± 0.7	2.78 ± 0.03	-	0.95	13	10	0.74
Supp. Fig. 11b-3	110 ± 5.3	-	2.86 ± 0.02	-	0.98	16	14	0.15
Supp. Fig. 12b	209 ± 10.8	-	3.01 ± 0.03	-	0.93	11	9	2.82
Supp. Fig. 12c	117 ± 9.8	-	3.08 ± 0.05	-	0.92	14	12	1.03
Supp. Fig. 12d	109 ± 3.1	-	2.96 ± 0.02	-	0.97	15	13	0.37
Supp. Fig. 20a-2	108 ± 8.1	-	0.34 ± 0.04	-	0.85	11	9	1.89
Supp. Fig. 21b-1	517± 7.3	-	0.75 ± 0.02	-	0.96	11	9	6.00
Supp. Fig. 21b-2	198 ± 16.3	-	1.69 ± 0.03	-	0.94	11	9	1.47
Supp. Fig. 26c	155 ± 9.1	-	3.13 ± 0.03	56.73 ± 3.67	0.96	15	12	1.53
Supp. Fig. 27b	903 ± 95.2	-	0.79 ± 0.03	331.75 ± 19.6	0.90	14	11	27.6

Supplementary Note 3: Displacement of bend contours at parallel g vectors

The proposed model describes the displacement of a bend contour arising from the rotation of $\mathbf{g}_{hkl}$ and the corresponding tilt of lattice planes associated to $\mathbf{g}_{hkl}$ at a dislocation core. Thus, the displacement of the bend contours constructed from a set of parallel $\mathbf{g}_{hkl}$, i.e. parallel sets of planes, should be equal as the tilt for these sets of parallel planes is the same. As a demonstration of this geometric principle, Supplementary Figure 10a shows approximately equal displacements of the $\mathbf{g}_{210}$ and $\mathbf{g}_{420}$ bend contours. The width of the bend contour for $\mathbf{g}_{420}$ is smaller than the width of the bend contour for $\mathbf{g}_{210}$. Additionally, a node appears at the split of bend contours for $\mathbf{g}_{420}$. Based on $\mathbf{B}$ = [010] assigned for dislocation-1 (Fig. 2), we note that $\mathbf{B}$ = [010] is approximately aligned with the dislocation line **u** for dislocation-1 in the local area where dislocation-1 cuts through the $\mathbf{g}_{420}$ bend contour. This observation points to a predominantly screw dislocation character at this position in the field of view, matching expectations for $\mathbf{g}_{hkl}\cdot\mathbf{B} = n$ for integer n with $n - 1$ nodes[3]. Based on this observation, the $\mathbf{B}_{screw}$ and $\mathbf{B}_{edge}$ components of the total Burgers vector $\mathbf{B}$ can be determined using the Cherns and Preston rule and box self-consistent approach (see Supplementary Figure 19).

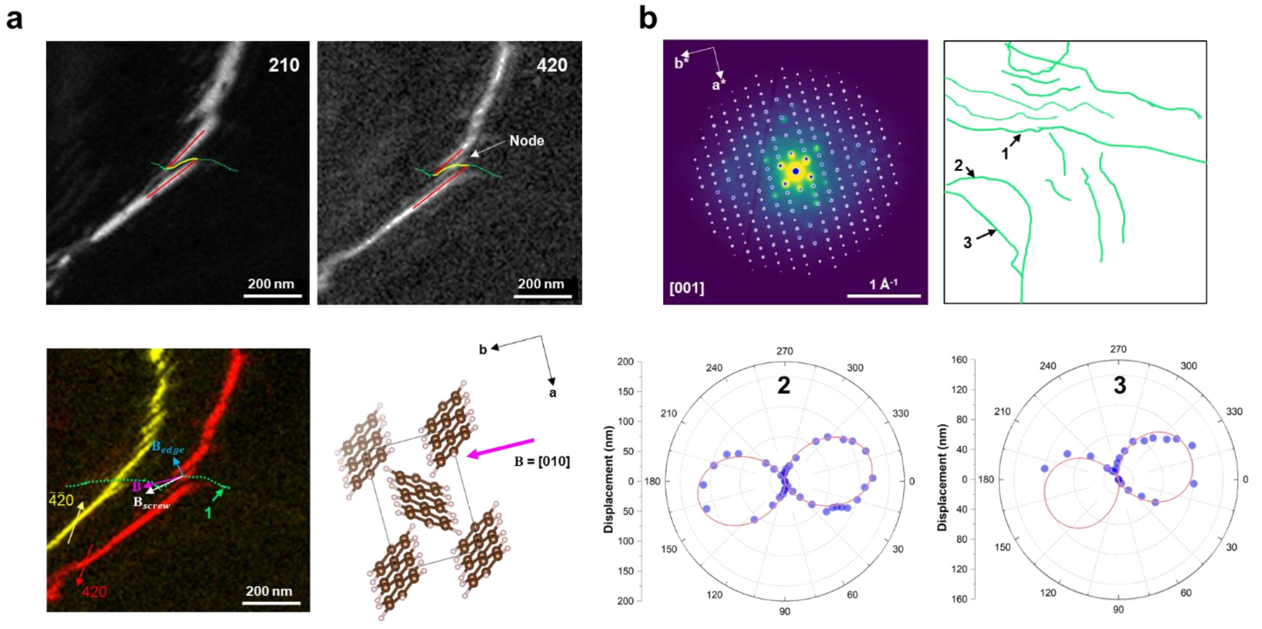

Supplementary Figure 10. Further analysis of dislocations in p-terphenyl in Fig. 2. a, Comparison between the displacements of the bend contours corresponding to parallel sets of planes, i.e. $\mathbf{g}_{210}$ and $\mathbf{g}_{420}$, at the dislocation. The node appears at the splitting of the bend contour for $\mathbf{g}_{420}$ where dislocation-1 is almost parallel to $\mathbf{B}$ = [010]. The signs of $\mathbf{B}_{screw}$ and $\mathbf{B}_{edge}$ components in the local area where dislocation-1 cuts through the $\mathbf{g}_{420}$ bend contour can be determined using Cherns-Preston rules and the total $\mathbf{B}$ identified via the polar plot analysis. **b**, Demonstration of this analysis approach on dislocation-2 and dislocation-3: (upper left) diffraction patterns (with on-zone simulated pattern overlaid in white) taken from the whole field of view, and (upper right) the mapping of the dislocations presented in the field of view. The polar plot of the bend contour displacements via azimuthal angle φ in diffraction space for dislocations-2 and -3 (bottom left and bottom right, respectively) show good fit to the simplified version of Equation S-3, similar to dislocation-1. The continuous bend contours, which are not affected by dislocations 2 and 3, are indexed as 200 and $\bar{2}00$ corresponding to the Burgers vector $\mathbf{B}$ = [010]. Modelling of the $\mathbf{B}$ direction in p-terphenyl unit cell viewing along [001] with adjusted in-plane rotation to match the orientation of the diffraction pattern from the experimental dataset.

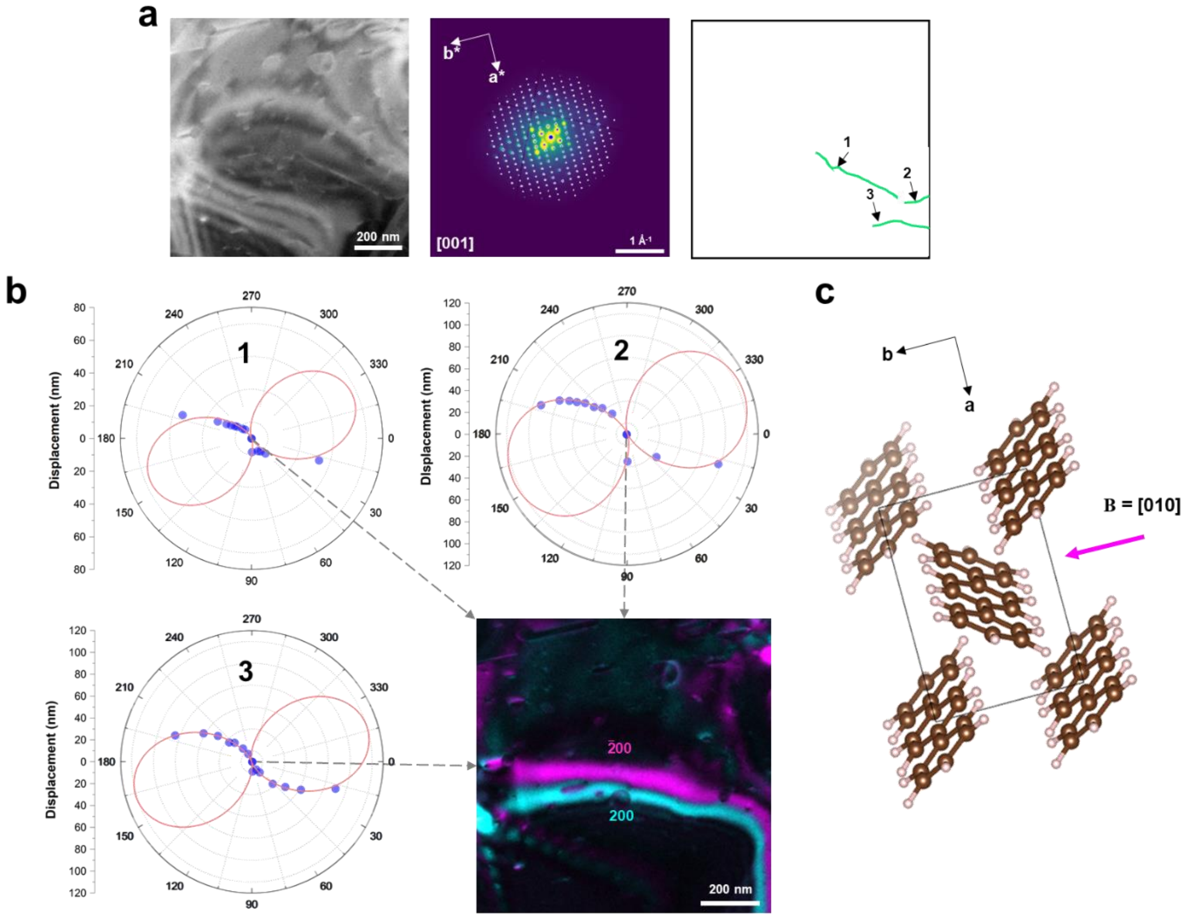

Supplementary Figure 11. Demonstration of the analysis approach on dislocations having only a few measurements of bend contour displacements. a, (Left) Annular dark field (ADF) image showing the pole bend contours and several in-plane and out-of-plane dislocations on the p-terphenyl film, (middle) corresponding diffraction pattern (with on-zone simulated pattern overlaid in white) indicating the slightly off zone axes of [001] which is in agreement with the pole bend contours slightly outside the field of view in the ADF image, and (right) in-plane dislocations are identified and presented by the green lines showing three small line dislocations. **b,** Polar plot of the bend contour displacement via azimuthal angle φ in diffraction space showing the non-complete plot for all three dislocations due to the limited field of view (1000x1000 nm) that does not allow to construct the observable bend contours outside the field of view. The fitting function following the simplified version of Equation S-3 is used to estimate the complete polar plot in these cases. The VDF image of the continuous bend contours can be constructed for the three dislocations showing the 200 and $\bar{2}00$ as the common continuous bend contours. The Burgers vector of all three dislocations, thus, follows $\mathbf{B}$ = [010]. **c,** Modelling of the $\mathbf{B}$ direction in p-terphenyl unit cell viewing along [001] direction with adjusted in-plane rotation to match the orientation of the diffraction pattern from experimental data set showing mixed-type for dislocation-1, pure screw for dislocation-2, and screw to mixed-type for dislocation-3.

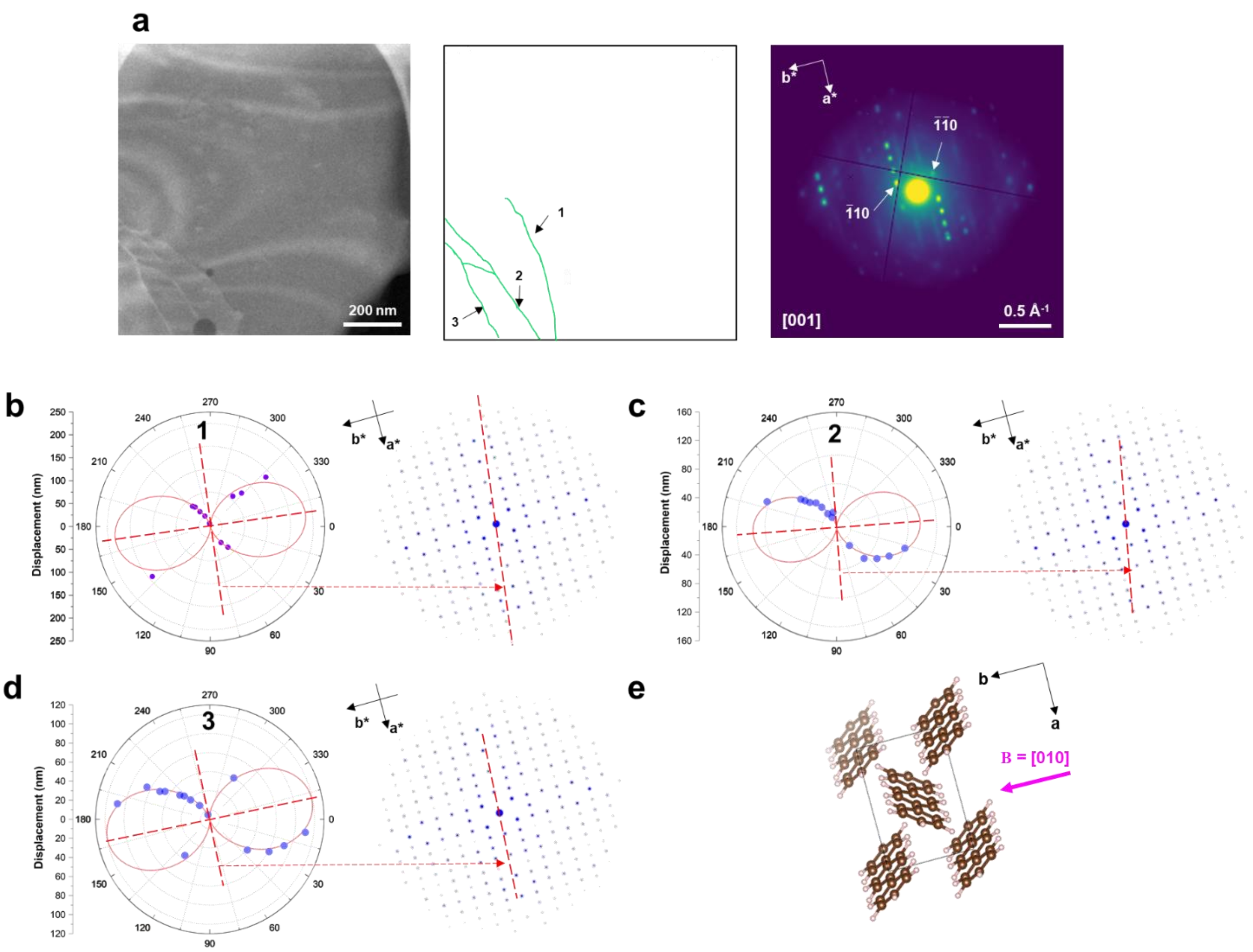

Supplementary Figure 12. Demonstration of the analysis approach for a p-terphenyl film tilted approximately 3.6° from the [001] zone axis. a, (Left) ADF image (1000x1000 nm) showing several bend contours crossing a dislocation network comprising of three major dislocation lines, (middle) in-plane dislocations are presented by the green lines and numbered, and (right) corresponding diffraction pattern indicating the substantial tilting away from the [001] zone axes as seen by the absence of the majority of diffraction spots/disks. **b,** Polar plot of the bend contour displacement via azimuthal angle φ constructed for dislocation-1 (with on-zone simulated pattern in blue added next to the plots). **c,** Polar plot of the bend contour displacement via azimuthal angle φ constructed for dislocation-2. **d,** Polar plot of the bend contour displacement via azimuthal angle φ constructed for dislocation-3. These polar plots show the non-complete plot and the absence of the diffraction vectors at the invisibility criterion condition for these dislocations. The fitting function following the simplified version of Equation S-3 estimates the complete polar plot that shows the invisibility criterion condition for dislocation-3 at $\mathbf{g}_{200}$ and $\mathbf{g}_{\bar{2}00}$. The Burgers vector of dislocation-3 is therefore $\mathbf{B}$ = [010]. However, the fitting function estimates the invisibility criterion condition for dislocation-1 at $\mathbf{g}_{\bar{1}\bar{1}\bar{1}0}$ and $\mathbf{g}_{1110}$ and dislocation-2 at $\mathbf{g}_{\bar{8}\bar{1}0}$ and $\mathbf{g}_{810}$, giving the Burgers vector [$u_\mathbf{B}v_\mathbf{B}$0] = [$1\overline{11}0$] and [$1\bar{8}0$], respectively which is 7.5° and 10.2° away from the common $\mathbf{B}$ = [010]. This deviation of dislocation-1 and dislocation-2 Burgers vectors may either arise from the residual uncertainties in bend contour measurements or substantial tilting of the sample from the [001] zone axes. **d,** Modelling of the two $\mathbf{B}$ directions in *p*-terphenyl unit cell viewing along [001] direction with adjusted in-plane rotation to match the orientation of the diffraction pattern from experimental data set showing dominant edge-type for dislocation-1 and mixed-type for dislocation-2 and dislocation-3.

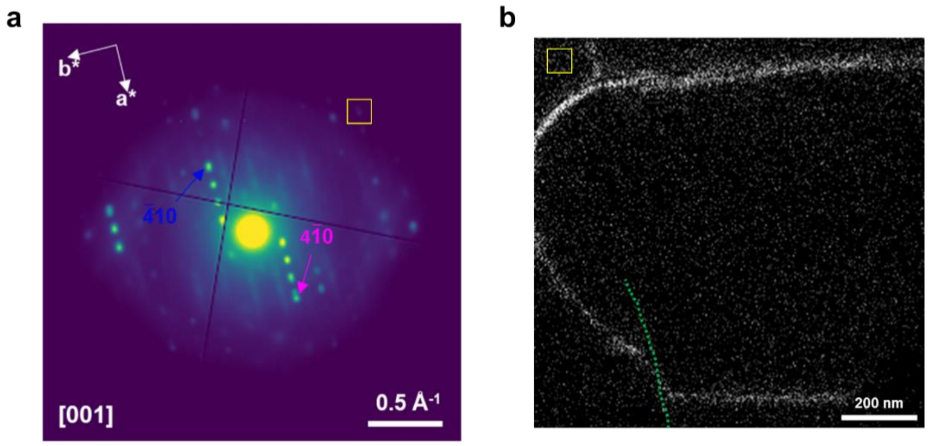

Supplementary Figure 13. Feasibility of high-angle scattering for bend contour analysis. a, A diffraction pattern from p-terphenyl (obtained at 8.8 e^{-} $Å^{-2}$) also presented in Supplementary Figure 12 highlighting the detection of scattering at 1.0837 $Å^{-1}$ (*d*-spacing 0.9227 Å) of sufficient quality for bend contour displacement analysis. **b**, The corresponding VDF image showing the bend contour at the Bragg disk highlighted in the yellow box in **a.** The green dashed line marks the dislocation line at the displacement in the bend contour.

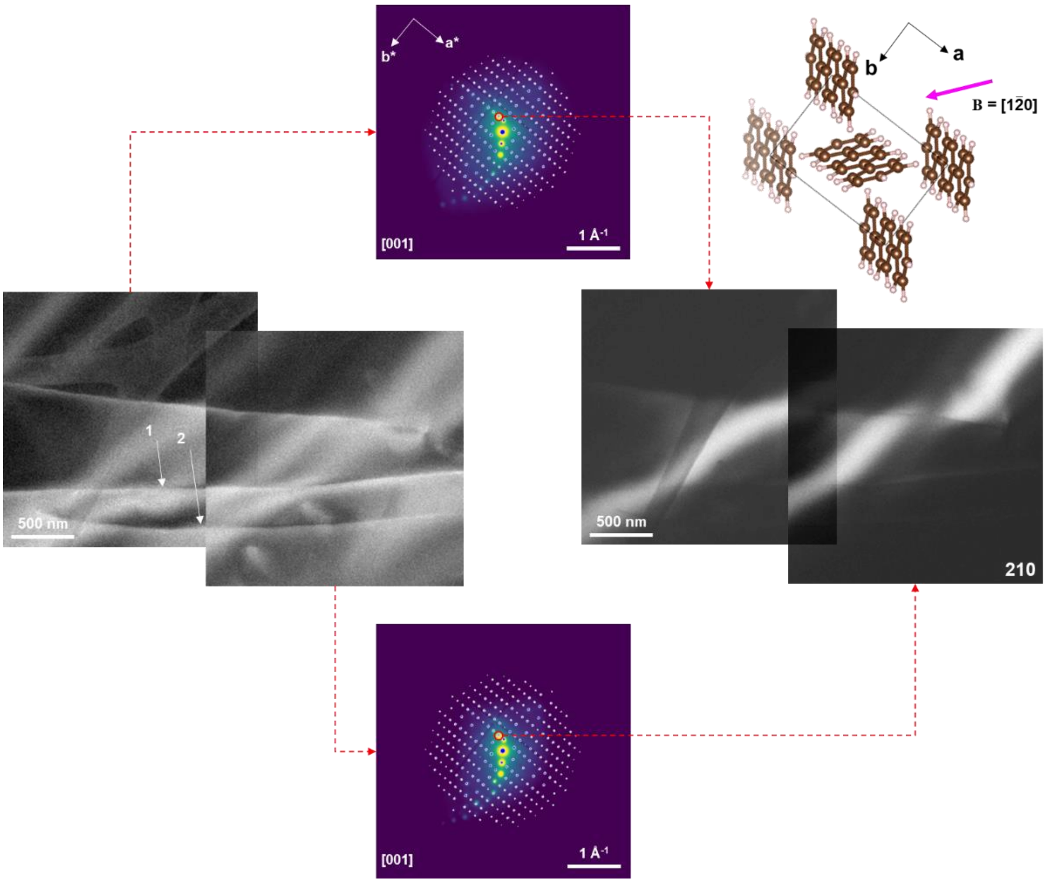

Supplementary Figure 14. SED measurement at two adjacent areas on the p-terphenyl film. ADF images showing the overlapping region between two SED datasets which can be used to merge two images. Corresponding diffraction patterns (with on-zone simulated pattern overlaid in white) show substantial tilting of the film away from the [001] zone axes. The VDF image constructed at the same $\mathbf{g}_{210}$ for these two datasets showing the shift of the 210 bend contour between two SED measurements. It suggests the re-orientation of the film under the scanning of the electron beam. The 210 bend contour shows no break on crossing dislocations-1 and -2 suggesting that these two dislocations have the same invisibility criterion condition at $\mathbf{g}_{210}$. The Burgers vector, thus, follows $\mathbf{B} = [1\bar{2}0]$. Modelling of the $\mathbf{B}$ direction (upper right) in p-terphenyl unit cell viewing along [001] direction with adjusted in-plane rotation to match the orientation of the diffraction pattern from experimental data set showing mixed-type for dislocation-1 and mixed-type for dislocation-1 and dislocation-2.

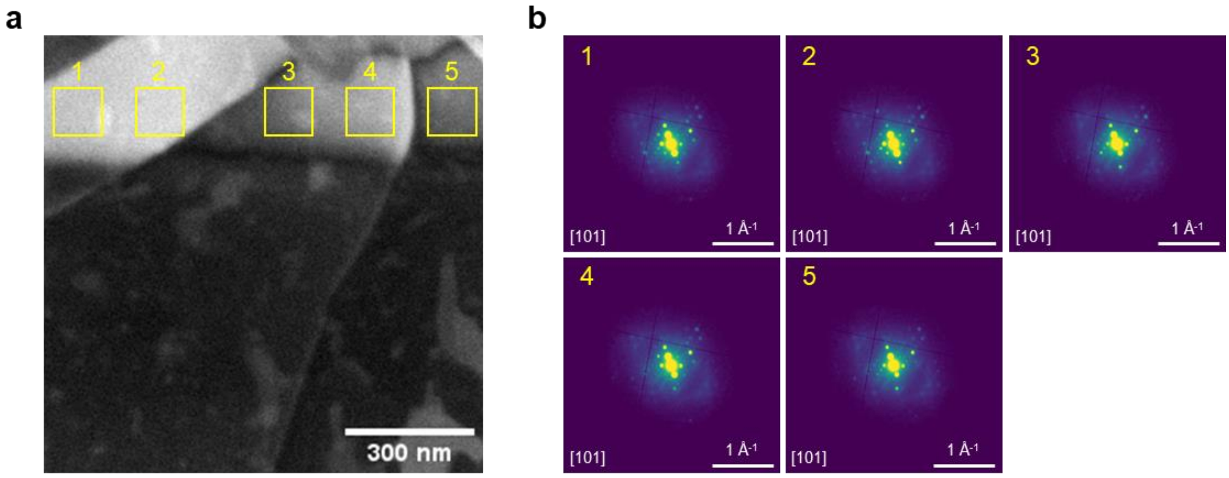

Supplementary Figure 15. Evaluation of diffraction changes along bright region passing linear features in a diffraction contrast image of the anthracene crystal presented in Figure 2. a, An ADF-STEM image of the anthracene crystal. Yellow squares mark regions of the image used for the extraction of diffraction patterns. **b**, Extracted diffraction patterns from regions marked in **a.** The diffraction patterns show a consistent arrangement of spots (and no in-plane rotation) and slowly varying intensities across the linear features. In contrast, in a planar defect (grain boundary or stacking fault), provided the two crystal orientations are sufficiently different, synchronized changes in the spot positions and/or intensities would occur across the defect. Planar defects such as 180° rotation twins, known in monoclinic crystals[4], give rise to distinct intensity distributions in each domain, though may have spots on a common reciprocal lattice vector basis. Likewise, grains with nearly identical orientations may not be readily distinguished from such a comparison of area-averaged diffraction patterns. As such, VDFs and diffraction patterns along the linear features are presented in Supplementary Figures 16-18 to further establish these features unambiguously as dislocations. Point defects would likely not introduce substantial changes in diffraction (unless sufficiently correlated to change the symmetry of the diffracting crystal which may introduced additional otherwise symmetry-forbidden diffraction spots).

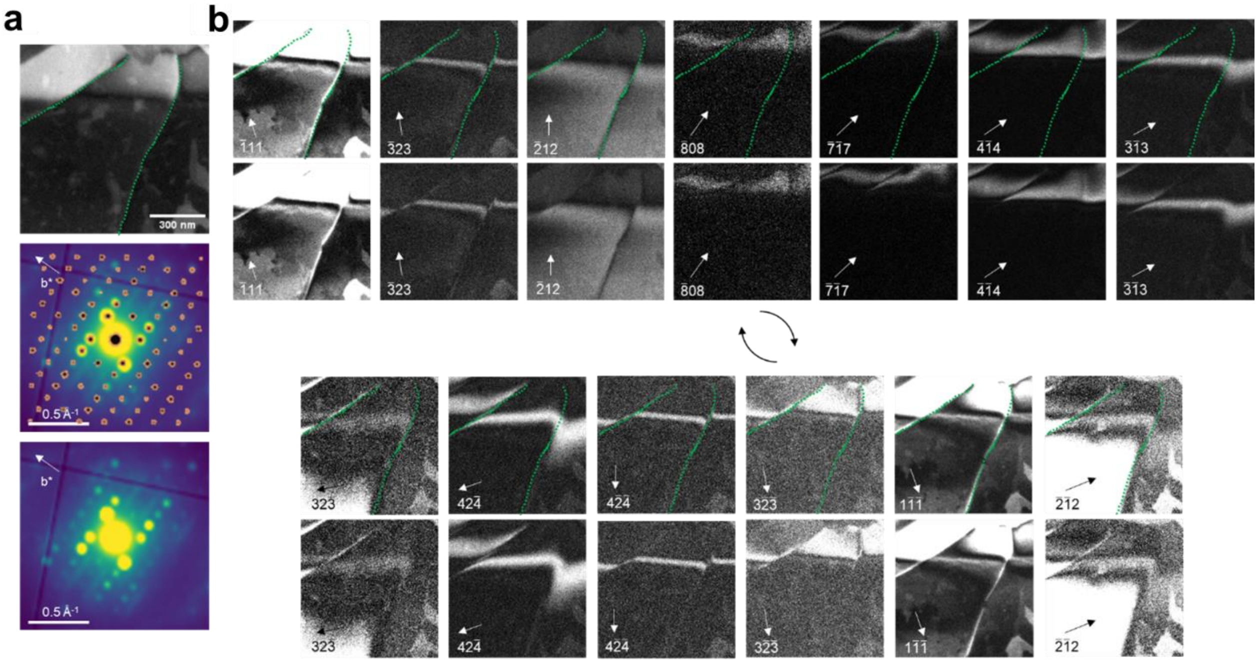

Supplementary Figure 16. Evaluation of the bend contour behaviours on passing linear features observed in diffraction contrast imaging of the anthracene crystal presented in Figure 2 and Supplementary Figure 15 above. a, (Top) Green lines marking the identified linear features on an ADF-STEM image, (middle) the corresponding average electron diffraction pattern overlaid with a simulated diffraction pattern for anthracene along the [101] zone axis, and (bottom) the average electron diffraction pattern repeated without the simulated pattern in overlay. **b**, A series of VDF images formed by placing virtual apertures at the recorded $\mathbf{g}_{hkl}$ positions showing the displacement of the bend contours on crossing the linear features. The Miller indices *hkl* and the vector direction are marked on each image. VDFs corresponding to several $\mathbf{g}_{hkl}$ reveal a 'twist' and node pattern characteristic of a screw component of a dislocation. This characteristic feature arises directly from the opposite displacement of planes across the helical screw dislocation core structure[3]. Screw component characteristics are observed for both linear features, confirming they are dislocations.

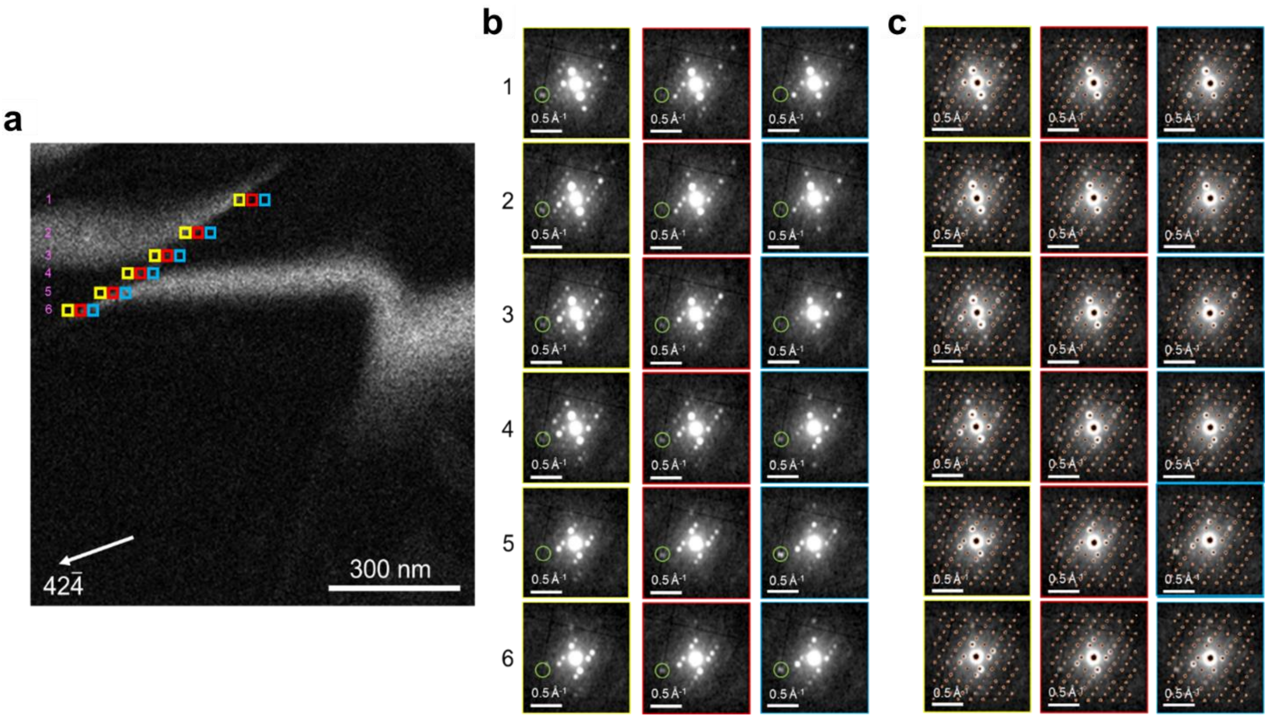

Supplementary Figure 17. Analysis of diffraction changes along and across dislocation 1 in the anthracene crystal presented in Figure 2. The designation (Dataset 2, dislocation-1) is given in Supplementary Figure 1. **a**, A VDF-STEM image constructed from an aperture placed around $\mathbf{g}_{42\bar{4}}$. . The white arrow marks the diffraction vector direction. The VDF shows the characteristic twist and node pattern of a screw component of a dislocation (see also Supplementary Figure 16). Coloured squares mark selected areas along the dislocation line where diffraction patterns were extracted. The colours (yellow, red, blue) denote the position to the left of the line, on the line, or to the right of the line. The vertical positions (rows) are numbered 1-6 from top to bottom. Each square corresponds to 5×5 pixels to achieve an acceptable signal-to-noise ratio in the reduced-area diffraction patterns. These areas highlight changes in the diffraction spot intensities along the twist and node pattern features. **b**-**c**, The corresponding diffraction patterns extracted from marked areas in **a**. In **b**, these patterns are displayed with an overlaid green circle marking the position of the VDF aperture. In **c**, these patterns are repeated with an overlaid simulated diffraction for anthracene along the [101] zone axis. The overlaid simulated patterns show a consistent arrangement of spots (and no in-plane rotation). Along the dislocation line and to the left of the line, the $\mathbf{g}_{42\bar{4}}$ spot is bright (row 1) and then fades (row 6). Along the dislocation line and to the right of the line the $\mathbf{g}_{42\bar{4}}$ spot is first faint (row 1) and then increases in intensity (row 6). The corresponding planes (*hkl*) are therefore tilted in opposite directions (up or down) as for the arrangement of planes with their surface normal vectors aligned with the screw component at a dislocation core. Other spots show a consistent arrangement of intensities with gradual changes in intensity across and along the line arising from continuous changes in orientation in the bent single crystal (likewise captured in the bend contours in the VDFs in Supplementary Figure 16).

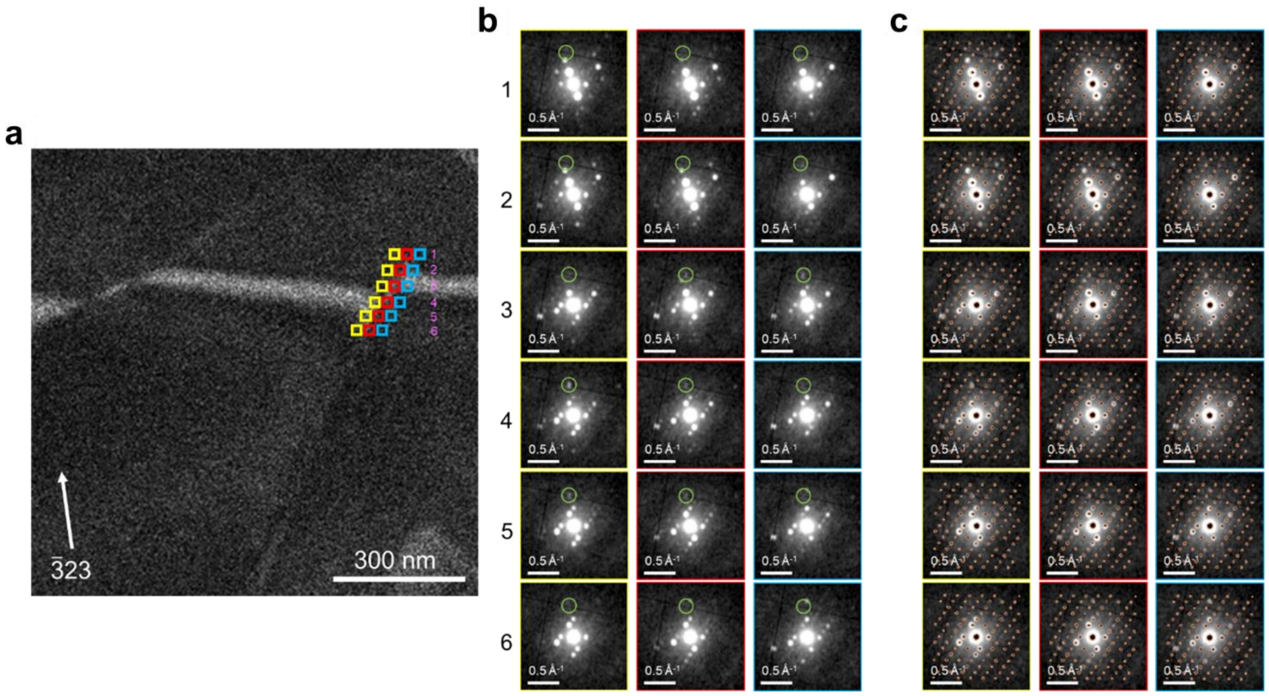

Supplementary Figure 18. Analysis of diffraction changes along and across dislocation-2 in the anthracene crystal presented in Figure 2. The designation (Dataset 2, dislocation-2) is given in Supplementary Figure 1. **a**, A VDF-STEM image constructed from an aperture placed around $\mathbf{g}_{\bar{3}23}$. The white arrow marks the diffraction vector direction. The VDF shows the characteristic twist and node pattern of a screw component of a dislocation (see also Supplementary Figure 16). The colours (yellow, red, blue) denote the position to the left of the line, on the line, or to the right of the line. The vertical positions (rows) are numbered 1-6 from top to bottom. Each square corresponds to 5×5 pixels to achieve an acceptable signal-to-noise ratio in the reduced-area diffraction patterns. These areas highlight changes in the diffraction spot intensities along the twist and node pattern features. **b**-**c**, The corresponding diffraction patterns extracted from marked areas in **a**. In **b**, these patterns are displayed with an overlaid green circle marking the position of the VDF aperture. In **c**, these patterns are repeated with an overlaid simulated diffraction for anthracene along the [101] zone axis. The overlaid simulated patterns show a consistent arrangement of spots (and no in-plane rotation). Along the dislocation line and to the left of the line, the $\mathbf{g}_{\bar{3}23}$ spot is faint (row 2-3) and then gains intensity (rows 4-5). Along the dislocation line and to right of the line, the $\mathbf{g}_{\bar{3}23}$ spot is first bright (rows 2-3) and then fades (row 4-5). The corresponding planes (*hkl*) are therefore tilted in opposite directions (up or down) as for the arrangement of planes with their surface normal vectors aligned with the screw component at a dislocation core. Other spots show a consistent arrangement of intensities with gradual changes in intensity across and along the line arising from continuous changes in orientation in the bent single crystal (likewise captured in the bend contours in the VDFs in Supplementary Figure 16).

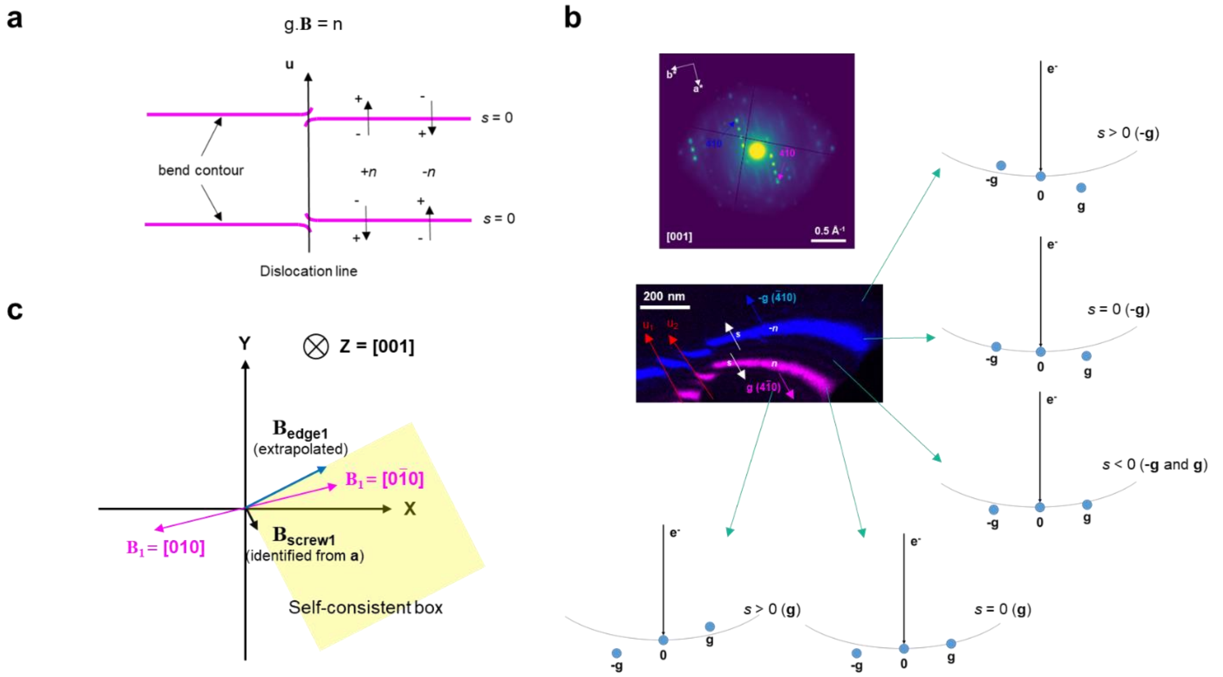

Supplementary Figure 19. Relative handedness of Burgers vector and its screw and edge components between individual dislocations. a, Application of Cherns and Preston rules for the splitting and twisting of bend contours[5] showing the determination of the signs of n in $\mathbf{g}_{hkl} \cdot \mathbf{B} = n$ condition. A 'twisting' of the bend contour highlights a screw character of dislocations which is due to the continuous change of the excitation error from negative to positive or vice versa for the diffracting planes on approaching the dislocation core (specific for screw-type components)[3]. **b,** Determination of excitation error (*s*) on either side of a bend contour (illustrated for p-terphenyl) using a pair of $\mathbf{g}_{hkl}$ and $-\mathbf{g}_{hkl}$ bend contours from the VDF image. **c,** Illustration of self-consistent box approach for the extrapolation of the relative handedness of edge components when the handedness of screw component (identified in **a**) and the direction of Burgers vector (identified by polar plot analysis) are known. The component vectors $\mathbf{B}_{\mathbf{edge}}$ and $\mathbf{B}_{\mathbf{screw}}$ are represented as components of $\mathbf{B}$ within the self-consistent region. The Cherns-Preston rules complement the geometric model by providing an independent validation of the Burgers vector's sign and direction, particularly in cases where the geometric fitting alone might be limited. This additional criterion allows for more robust and accurate determination of Burgers vector. By using the approaches in **a, b,** and **c**, we can determine the sign, and in specific cases – the magnitude, of a Burgers vector and the relative handedness of its edge and screw components for individual dislocations.

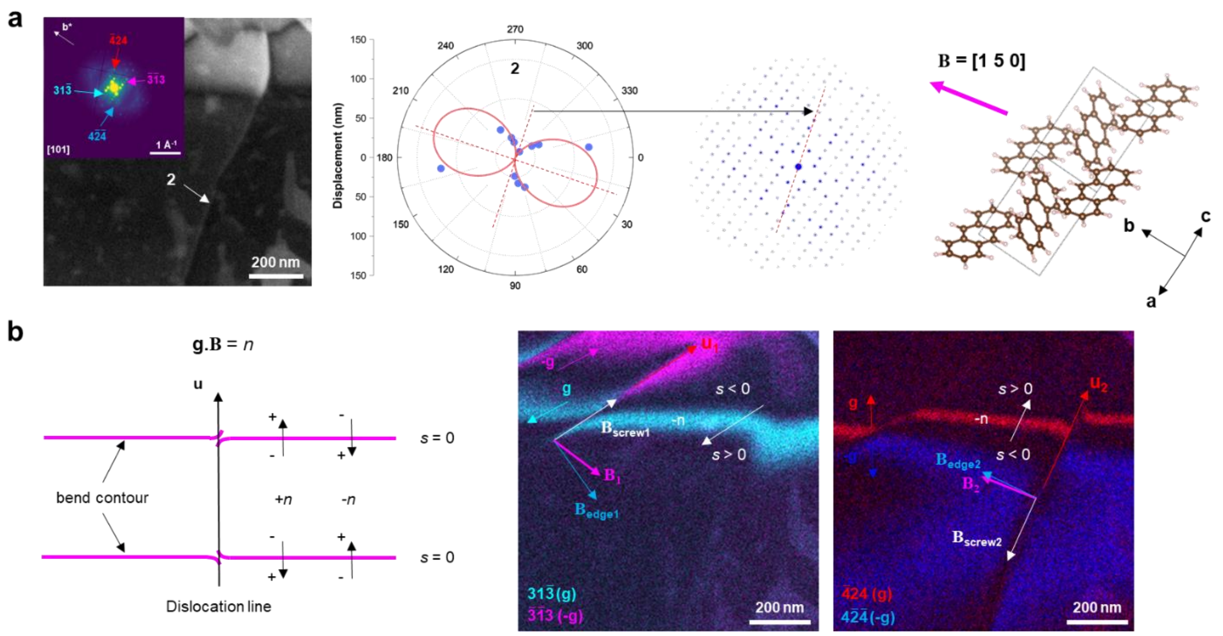

Supplementary Figure 20. Analysis of a second dislocation in the anthracene film in Figure 2. a, (Left) ADF-STEM image with average electron diffraction pattern inset, (middle) polar plot of bend contour breaks at a dislocation line and accompanying fit displayed on a simulated diffraction pattern, and (right) visualisation of the crystal structure with determined Burgers vector shown. The incomplete polar plot of the bend contour displacement on crossing dislocation-2 via azimuthal angle φ shows good fit to the simplified version of Equation S-3, similar to dislocation-1. There is an absence of the diffraction vectors at the invisibility criterion condition for this dislocation and the fitting function estimates the complete polar plot indicating such a condition at ~ $\mathbf{g}_{5\bar{1}\bar{5}}$ and $\mathbf{g}_{\bar{5}15}$ for dislocation-2. The Burgers vector of this dislocation, determined using equation S-7 for the monoclinic anthracene crystal where $\mathbf{a} \cdot \mathbf{b} \neq 0$, follows $\mathbf{B} = [u_\mathbf{B}, v_\mathbf{B}, w_\mathbf{B}]$ with $u_\mathbf{B}$ = -0.16, $v_\mathbf{B}$ = -1.00, $w_\mathbf{B}$ = 0.04 which is approximately along $[u_\mathbf{B} v_\mathbf{B} 0] = [\bar{1}\bar{5}0]$ or $[150]$ (3.6° from perpendicular to [101] and 3.6° from extracted $\mathbf{B}$, 1.5 standard errors for a standard error of 0.04 radians). Modelling of the $\mathbf{B}$ direction in anthracene unit cell viewing along [101] direction with adjusted in-plane rotation to match the orientation of the diffraction pattern from the experimental data set showing mixed-type dislocation for dislocation-2 but with an edge component that is predominant. **b**, Relative handedness of screw components between dislocation-1 and dislocation-2 in anthracene film: (Left) application of Cherns and Preston rules for the splitting and twisting of bend contours, (middle) determination of excitation error (*s*), $\mathbf{B}$, $\mathbf{B}_{screw}$ and $\mathbf{B}_{edge}$ directions for dislocation-1, and (right) determination of excitation error (*s*) and $\mathbf{B}$, $\mathbf{B}_{screw}$ and $\mathbf{B}_{edge}$ directions for dislocation-2. Following the determination of excitation error *s* and the Cherns and Preston rule for $\mathbf{B}_{screw}$ component, the condition of $\mathbf{g}_{hkl} \cdot \mathbf{B}_{screw1} = -n$ is obtained for dislocation-1 with $\mathbf{g}_{31\bar{3}}$. By applying the same procedure for dislocation-2, the condition $\mathbf{g}_{hkl} \cdot \mathbf{B}_{screw2} = -n$ is also obtained with $\mathbf{g}_{\bar{4}24}$. Comparing the direction of the Burgers vectors and the components shows the opposite handedness of dislocation-1 and -2 in the anthracene film, possibly pinned by features at the top right of the field of view in **a**.

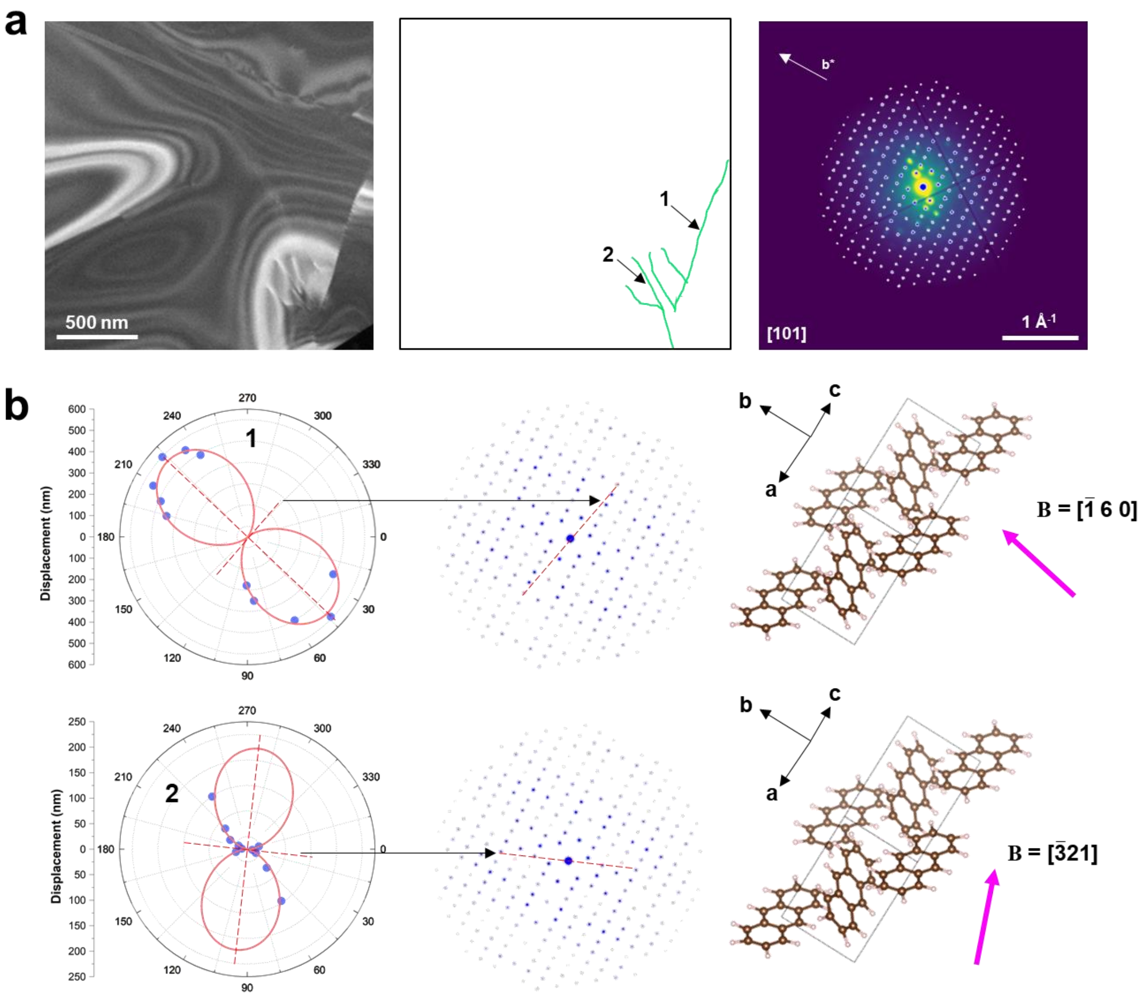

Supplementary Figure 21. Application of the analysis approach on the anthracene film prepared by spin coating. a, (Left) ADF image showing breaks in bend contours on crossing a dislocation network, (middle) in-plane dislocations are presented by the green lines and are numbered, and (right) corresponding diffraction pattern (with on-zone simulated pattern overlaid in white) indicating the substantial tilting of the film away from the [101] zone axes as seen by the absence of the majority of diffraction spots/disks shown in the overlaid zone axis simulation (white spots). **b,** (Left) Polar plot of the bend contour displacement via azimuthal angle φ constructed for dislocation-1 and -2 showing an incomplete plot and the absence of the diffraction vectors at the invisibility criterion condition for these dislocations. The fitting function following the simplified version of Equation S-3 provides the complete polar plot that shows the invisibility criterion condition at $\mathbf{g}_{61\bar{6}}$ and $\mathbf{g}_{\overline{616}}$ for dislocation-1 and $\mathbf{g}_{12\bar{1}}$ and $\mathbf{g}_{\overline{121}}$ for dislocation-2 after indexation to the on-zone simulated diffraction pattern (middle). (Right) The Burgers vector for dislocation-1 follows $\mathbf{B} = [u_\mathbf{B}, v_\mathbf{B}, w_\mathbf{B}]$ with $u_\mathbf{B} = -0.13$, $v_\mathbf{B} = 1.00$, $w_\mathbf{B} = 0.04$ which is approximately along $[u_\mathbf{B} v_\mathbf{B} 0] = [\bar{1}60]$ (3.1° from perpendicular to [101] and 3.1° from extracted $\mathbf{B}$, 2.5 standard errors for a standard error of 0.02 radians). The Burgers vector for dislocation-2 follows $\mathbf{B} = [u_\mathbf{B}, v_\mathbf{B}, w_\mathbf{B}]$ with $u_\mathbf{B} = -0.13$, $v_\mathbf{B} = -1.00$, $w_\mathbf{B} = 0.04$ which is approximately along $[\bar{3}21]$ (2.4° from perpendicular to [101] and 2.4° from extracted $\mathbf{B}$, 1.3 standard errors for a standard error of 0.03 radians). This dislocation-2 Burgers vector has not been reported previously nor would appear to be readily expected; it may either arise from the method of preparation, proximity to other dislocations, or from limitations in the dataset due to residual uncertainties in bend contour measurements or sample orientation effects.

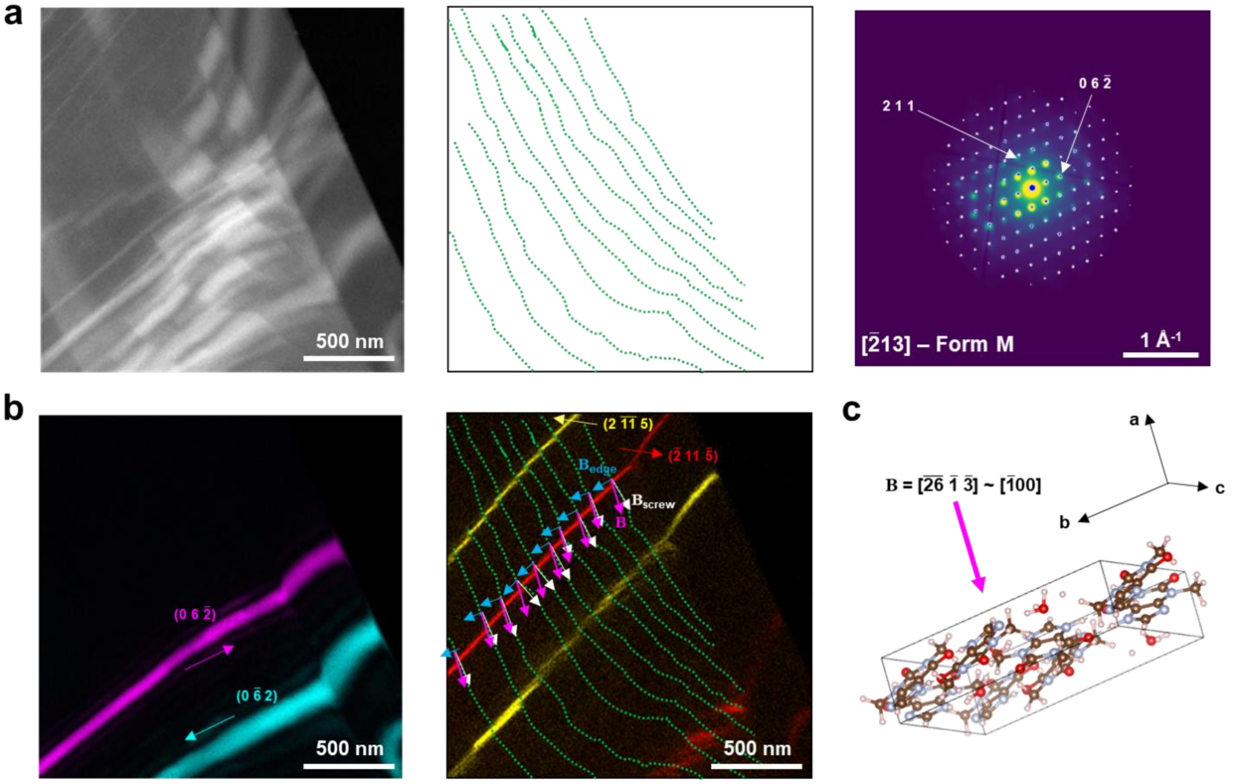

Supplementary Figure 22. Analysis of dislocation network in theophylline monohydrate (Form M) prepared by the solvent evaporation from nitromethane:water solution. a, (Left) ADF image showing several bend contours on crossing a large dislocation network across the field of view. Individual dislocations in the dislocation network are presented by the green lines. Corresponding diffraction pattern (with on-zone simulated pattern overlaid in white) showing the monohydrate theophylline film crystallised on the [$\bar{2}$13] zone axis. **b,** VDF images of the bend contour pairs at different $\mathbf{g}_{hkl} \cdot \mathbf{B}$ conditions. VDF image of the bend contour pair constructed from $\mathbf{g}_{06\bar{2}}$ and $\mathbf{g}_{0\bar{6}2}$ showing the continuous bend contours on crossing several dislocations which highlights the invisibility criterion condition at these two diffraction vectors. VDF image of the bend contour pair constructed from $\mathbf{g}_{2\bar{1}\bar{1}5}$ and $\mathbf{g}_{\bar{2}11\bar{5}}$ showing the break and twist of bend contours at individual dislocations. The Burgers vector for individual dislocations in the dislocation network follows $\mathbf{B}$ = [$u_{\mathbf{B}}$, $v_{\mathbf{B}}$, $w_{\mathbf{B}}$] with $u_{\mathbf{B}}$ = 1.00, $v_{\mathbf{B}}$ = 0.04, $w_{\mathbf{B}}$ = 0.12 or $\mathbf{B}$ = [26 1 3] or [$\overline{26}$ $\bar{1}$ $\bar{3}$]. This unlikely high index vector aligns closely with the projection of the [100] or [$\bar{1}$00] directions. Applying the Cherns and Preston rule and the self-consistent box approach, the direction of the Burgers vector and their screw and edge components can be determined with the condition $\mathbf{g}_{hkl} \cdot \mathbf{B} = \mathbf{1}$ for $\mathbf{g}_{\bar{2}11\bar{5}}$, therefore, the Burgers vector was assigned as $\mathbf{B}$ = ½[$\bar{1}$00]. **c,** Modelling of the $\mathbf{B}$ direction in theophylline form M unit cell viewing along [$\bar{2}$13] with adjusted in-plane rotation to match the orientation of the diffraction pattern from the experimental data set.

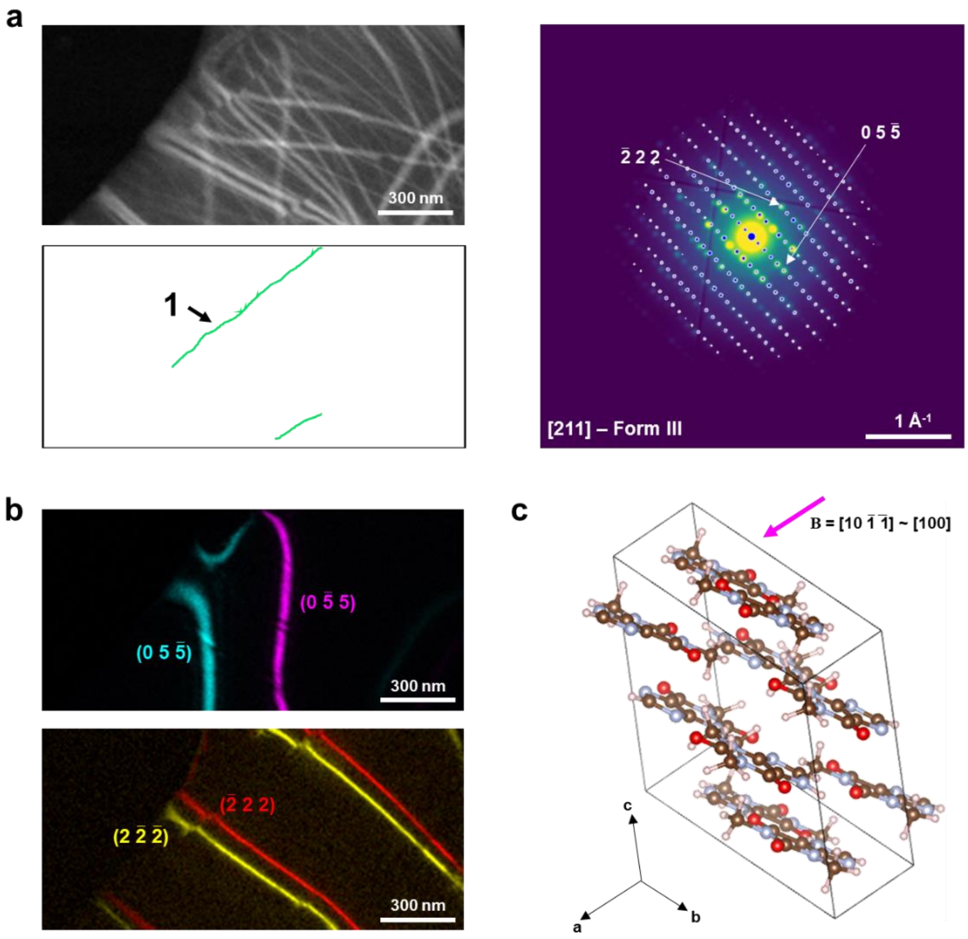

Supplementary Figure 23. Analysis of individual dislocations in metastable theophylline (Form IIIb) particle. Data acquisition was carried out under liquid nitrogen cooling with a cryo-holder. **a,** ADF image showing bend contour network on crossing dislocations at some areas of the film. Individual dislocations are presented by green lines at which the bend contours show breaking in the ADF image. Corresponding diffraction pattern (with on-zone simulated pattern overlaid in white) showing theophylline From III film viewed along the [211] zone axis. **b,** VDF images of the bend contour pairs at different $\mathbf{g}_{hkl} \cdot \mathbf{B}$ conditions. VDF image of the bend contour pair constructed from $\mathbf{g}_{05\bar{5}}$ and $\mathbf{g}_{0\bar{5}5}$ showing the continuous bend contours on crossing dislocations which highlights the invisibility criterion condition at these two diffraction vectors. VDF image of the bend contour pair constructed from $\mathbf{g}_{2\bar{2}\bar{2}}$ and $\mathbf{g}_{\bar{2}22}$ showing the break of bend contours at individual dislocations. The Burgers vector for individual dislocations follows $\mathbf{B} = [u_{\mathbf{B}}, v_{\mathbf{B}}, w_{\mathbf{B}}]$ with $u_{\mathbf{B}} = -1.00$, $v_{\mathbf{B}} = 0.10$, $w_{\mathbf{B}} = 0.10$ or $\mathbf{B} = [\overline{10}\ 1\ 1]$ or $[10\ \bar{1}\ \bar{1}]$. This unlikely high index vector aligns closely with the projection of the $[100]$ or $[\bar{1}00]$ directions. These directions are parallel to the dislocation-1 direction. Thus, dislocation-1 exhibits predominantly screw-type character. Using Cherns and Preston rule for the bend contour associated with $\mathbf{g}_{2\bar{2}\bar{2}}$, the condition $\mathbf{g}_{hkl} \cdot \mathbf{B} = \mathbf{2}$ can be established, and therefore the Burgers vector was assigned as $\mathbf{B} = [100]$. **c,** Modelling of the $\mathbf{B}$ direction in theophylline form IIIb unit cell viewed along [211] with adjusted in-plane rotation to match the orientation in the diffraction pattern from the experimental data set.

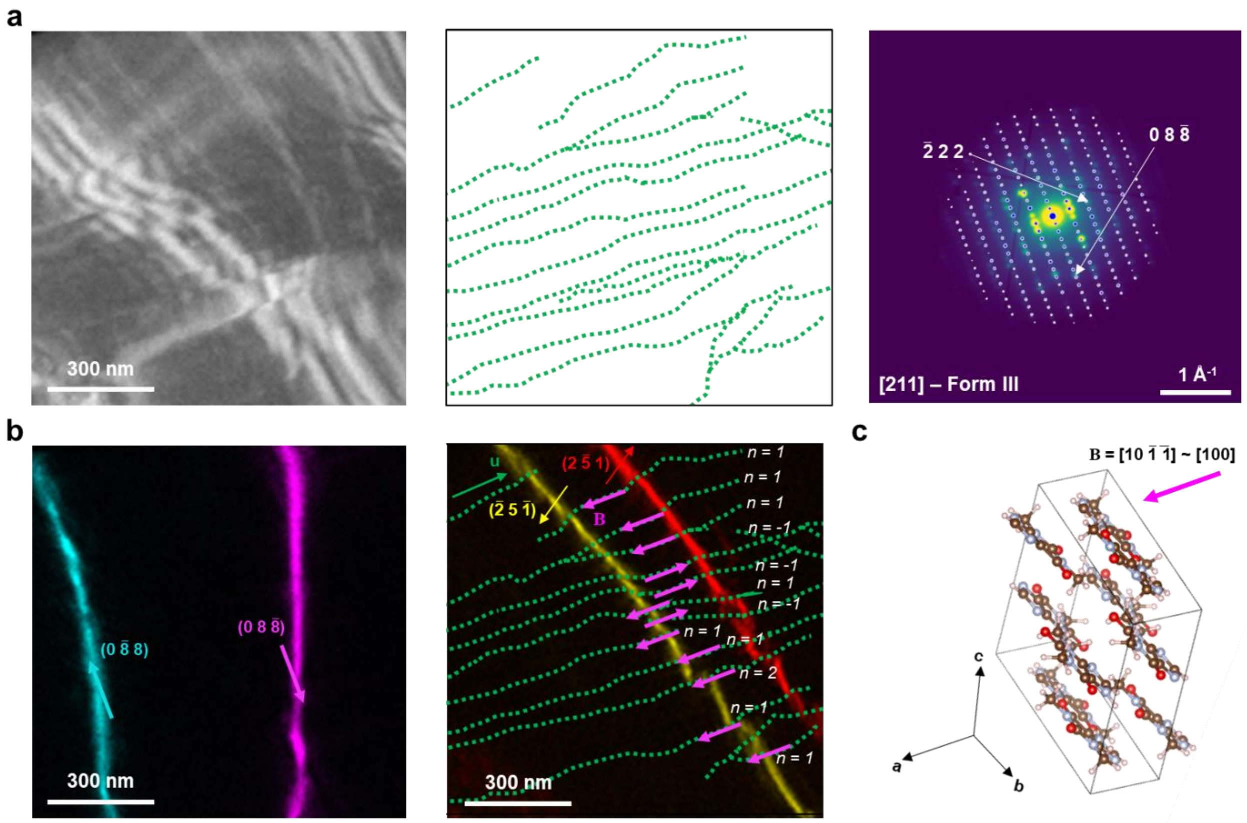

Supplementary Figure 24. Analysis of dislocation network in metastable theophylline (Form IIIb) particle. Data acquisition was carried out under liquid nitrogen cooling with a cryo-holder. **a,** ADF image showing bend contour network on crossing dislocations at some areas of the film. Individual dislocations are presented by green lines at which the bend contours show breaking in the ADF image. Corresponding diffraction pattern (with on-zone simulated pattern overlaid in white) showing theophylline From III film viewed along the [211] zone axis. **b,** VDF images of the bend contour pairs at different $\mathbf{g}_{hkl} \cdot \mathbf{B}$ conditions. VDF image of the bend contour pair constructed from $\mathbf{g}_{08\bar{8}}$ and $\mathbf{g}_{0\bar{8}8}$ showing the continuous bend contours on crossing dislocations which highlights the invisibility criterion condition at these two diffraction vectors. VDF image of the bend contour pair constructed from $\mathbf{g}_{2\bar{5}1}$ and $\mathbf{g}_{\bar{2}5\bar{1}}$ showing the break and twist of bend contours at individual dislocations. The Burgers vector for individual dislocations follows $\mathbf{B} = [u_\mathbf{B}, v_\mathbf{B}, w_\mathbf{B}]$ with $u_\mathbf{B} = -1.00$, $v_\mathbf{B} = 0.10$, $w_\mathbf{B} = 0.10$ or $\mathbf{B} = [\overline{10}\ 1\ 1]$ or $[10\ \bar{1}\ \bar{1}]$. This unlikely high index vector aligns closely with the projection of the $[100]$ or $[\bar{1}00]$ directions. These directions are parallel to the dislocation direction in the network. Thus, the dislocations exhibit predominantly screw-type characters. Using Cherns and Preston rule for the bend contour associated with $\mathbf{g}_{\bar{2}5\bar{1}}$, the condition $\mathbf{g}_{hkl} \cdot \mathbf{B} = n$ with n = -1, 1 or 2 can be established for some dislocations, and therefore the Burgers vector can be assigned as $\mathbf{B} = ½[100]$, $½[\bar{1}00]$ or $[\bar{1}00]$. **c,** Modelling of the $\mathbf{B}$ direction in theophylline form IIIb unit cell viewed along [211] with adjusted in-plane rotation to match the orientation in the diffraction pattern from the experimental data set.

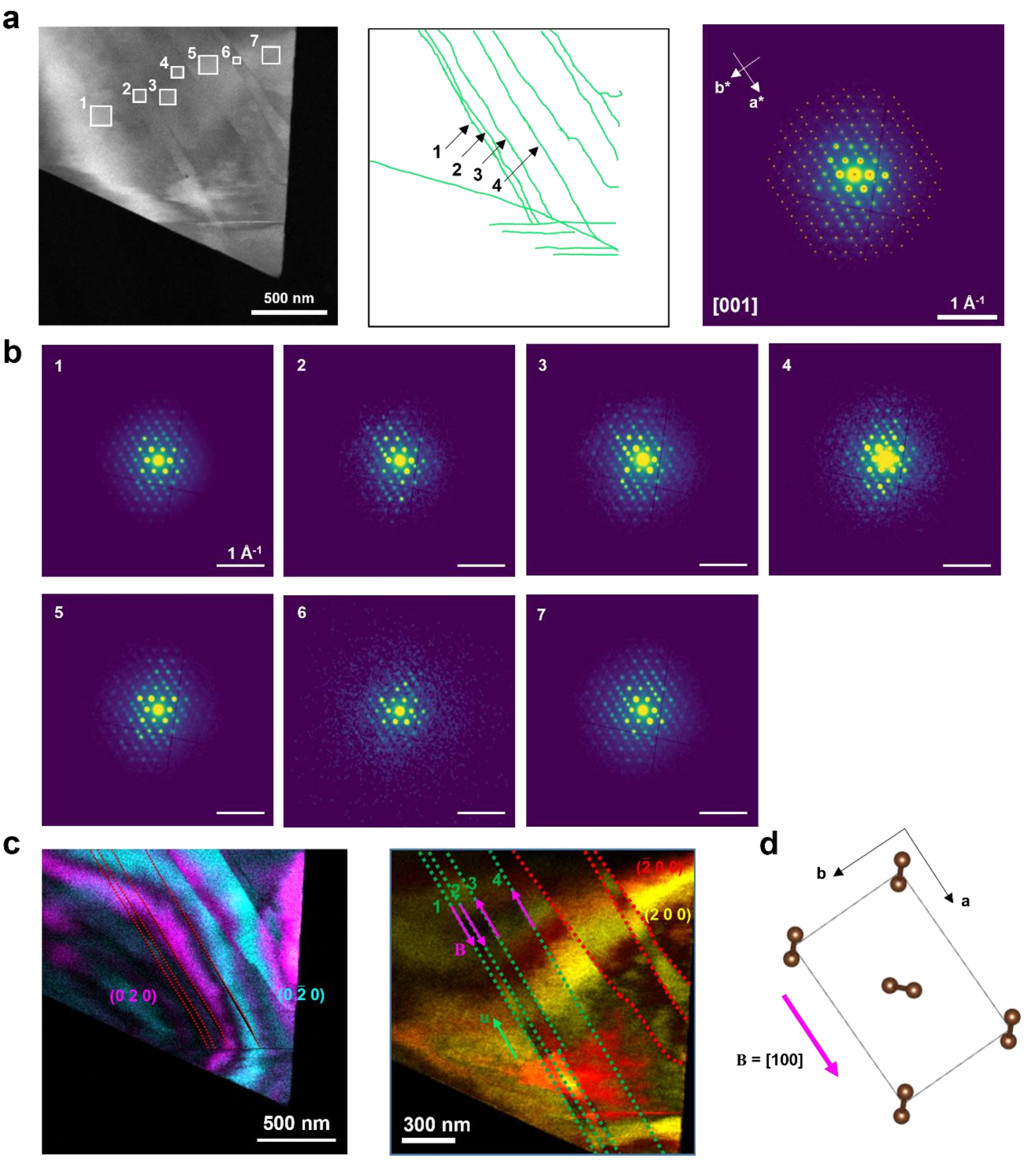

Supplementary Figure 25. Analysis of dislocation network in a 1-triacontanol wax crystal. a, ADF image showing dislocation lines and the shift of a bend contour on crossing a dislocation. Several dislocations are presented by green lines, showing the dense network across the crystal. Corresponding diffraction pattern (with on-zone simulated pattern overlaid in white) showing the wax crystal aligns on the [001] zone axis. 1-triacontanol crystals exhibit equivalent polyethylene-like packing as observed in *n*-hentriacontane and other long-chain waxes when viewed along the chains[6]. The *Pnam* polyethylene unit cell[7-8] was used for simulation and indexation. The *b*-axis in the *Pnam* polyethylene unit cell corresponds to the *a*-axis in the *n*-hentriacontane *Pbcm* cell in Figure 3h (and the *a*-axis in the *Pnam* polyethylene unit cell corresponds to the *b*-axis). **b,** Electron diffraction patterns taken at different areas between the dislocation lines showing the same in-plane orientation suggesting that these areas are not from different domains, although from diffraction pattern inspection alone there is a possibility these are grain boundaries with 180° relative in-plane rotation between grains. **c,** VDF images of the bend contour pairs at different $\mathbf{g}_{hkl} \cdot \mathbf{B}$ conditions**.** VDF image of the bend contour pairs showing continuous bend contours on crossing dislocation-1, -2, -3, and -4 at $\mathbf{g}_{020}$ and $\mathbf{g}_{0\bar{2}0}$ and a break in the bend contours at $\mathbf{g}_{200}$ and $\mathbf{g}_{\bar{2}00}$. On balance, these observations point to these features as dislocations. The Burgers vector for these dislocations follows $\mathbf{B}$ = [100]. However, for other line features (marked in red), the magnitude of bend contours break remains approximately similar for different **g** vectors, suggesting that these lines are more likely to be grain boundaries. **c,** Modelling of the $\mathbf{B}$ direction using the polyethylene unit cell for indexation viewed along [001] with adjusted in-plane rotation to match the orientation in the diffraction pattern from the experimental data set.

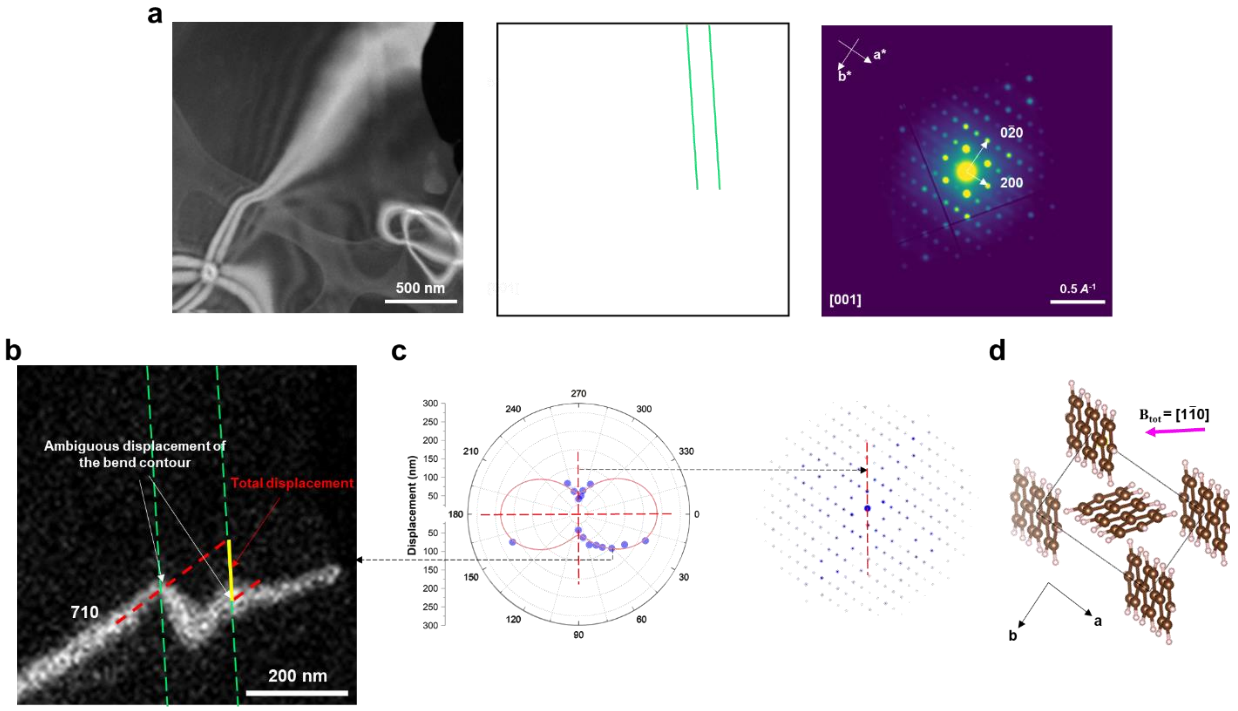

Supplementary Figure 26. Adjacent, parallel dislocation lines in p-terphenyl. This dataset was taken at 200 kV. **a,** ADF image of the p-terphenyl film showing several pole bend contours within the field of view. One of the bright bend contour intersects with two parallel dislocation lines as presented by the green lines (middle panel). The corresponding diffraction pattern for the whole field of view is shown on the right, indicating that the film is oriented along the [001] zone axis. **b**, VDF image of the g_{710} bend contour highlighting the limited separation and resulting challenge for reliable identification of bend contour displacements for each individual dislocation line. The total displacement of the bend contour on crossing both dislocations was measured to examine the information on the combined pair of dislocations. **c**, A polar plot showing total displacement of the bend contours on crossing both dislocations via azimuthal angle φ (with on-zone simulated pattern added next to the plot). The fitting function (equation 1) can be modified with an additional parameter *D*: $f(\varphi) = A\arctan(B\cos^2(\varphi - C)) + D$ to capture the observed response. The resulting plot and fit does not reach zero, possibly due to the contribution (sum) of two dislocations with different Burgers vectors or due to deviations from a linear and elastic response between the two dislocations. Estimation of quasi-invisibility criterion condition using the local minimum of the constructed polar plot and the simulated diffraction pattern showing the diffraction vector $\mathbf{g}_{110}$ and $\mathbf{g}_{\bar{1}\bar{1}0}$. One possible explanation is that the Burgers vectors of the two dislocations sum to $\mathbf{B}_{tot}$ = [1$\bar{1}$0]. **d,** The $\mathbf{B}_{tot}$ direction in p-terphenyl unit cell viewing along [001] direction with adjusted in-plane rotation to match the orientation of the diffraction pattern.

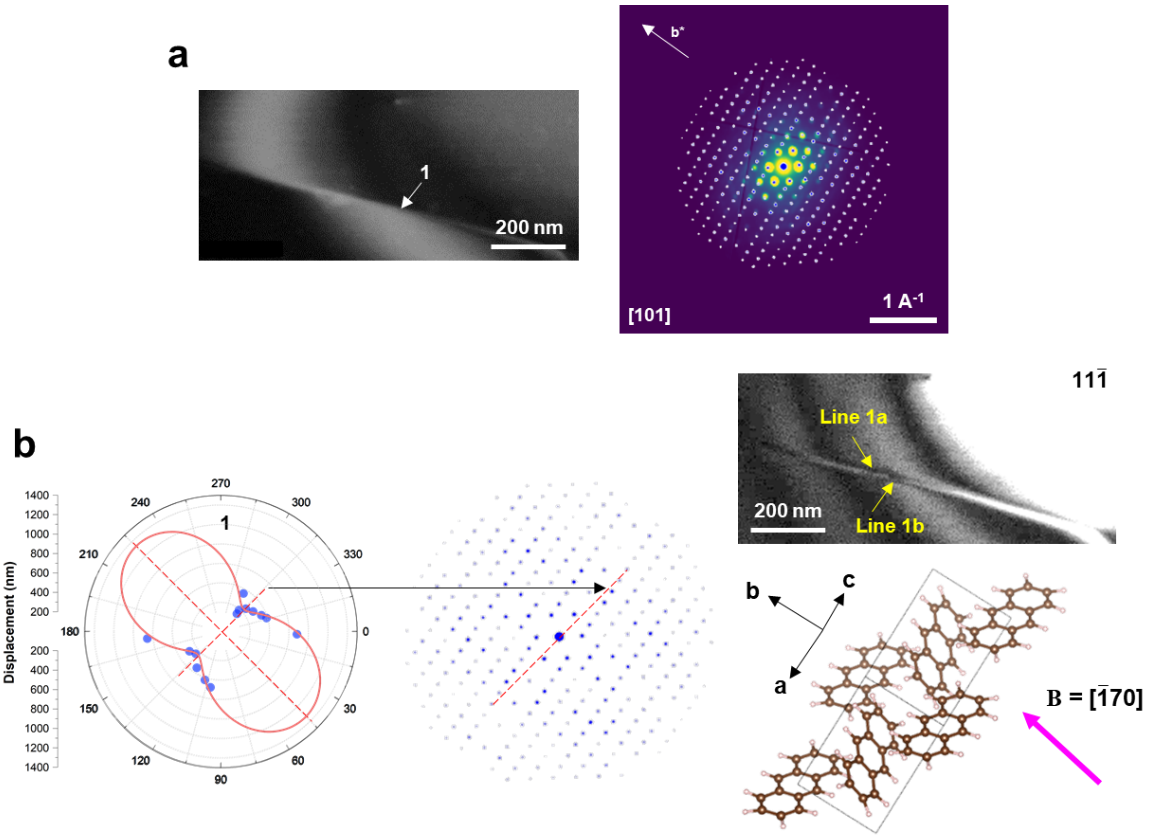

Supplementary Figure 27. Adjacent, parallel dislocation lines in anthracene. a, ADF image showing the shifting of a bright bend contour on crossing dislocation-1 and the corresponding diffraction pattern (with on-zone simulated pattern overlaid in white) indicating the film near the [101] zone axis. **b,** A polar plot of the bend contour displacement as a function of azimuthal angle φ constructed for dislocation-1 with on-zone simulated pattern added next to the plot. Fitting was carried out using a modified form of equation 1 with an additional fitting parameter *D* (see also Supplementary Figure 26). (Right) VDF image constructed from $\mathbf{g}_{11\bar{1}}$ shows two dislocation lines in very close proximity with near-parallel dislocation lines. The polar plot shows a quasi-invisibility criterion condition at ~ $\mathbf{g}_{\bar{7}\bar{1}7}$ and $\mathbf{g}_{71\bar{7}}$ for the combined dislocations. The extracted Burgers vector follows $\mathbf{B}_{tot}$ = [$u_\mathbf{B}$, $v_\mathbf{B}$, $w_\mathbf{B}$] with $u_\mathbf{B}$ = -0.11, $v_\mathbf{B}$ = 1.00, $w_\mathbf{B}$ = 0.03 which is approximately along [$u_\mathbf{B}v_\mathbf{B}$0] = [$\bar{1}$70] (2.6° from perpendicular to [101] and 2.6° from the extracted **B**, 1.5 standard errors for a standard error of 0.03 radians). Modelling of the $\mathbf{B}_{tot}$ direction in anthracene unit cell viewing along [101] with adjusted in-plane rotation to match the orientation of the diffraction pattern from experimental dataset.

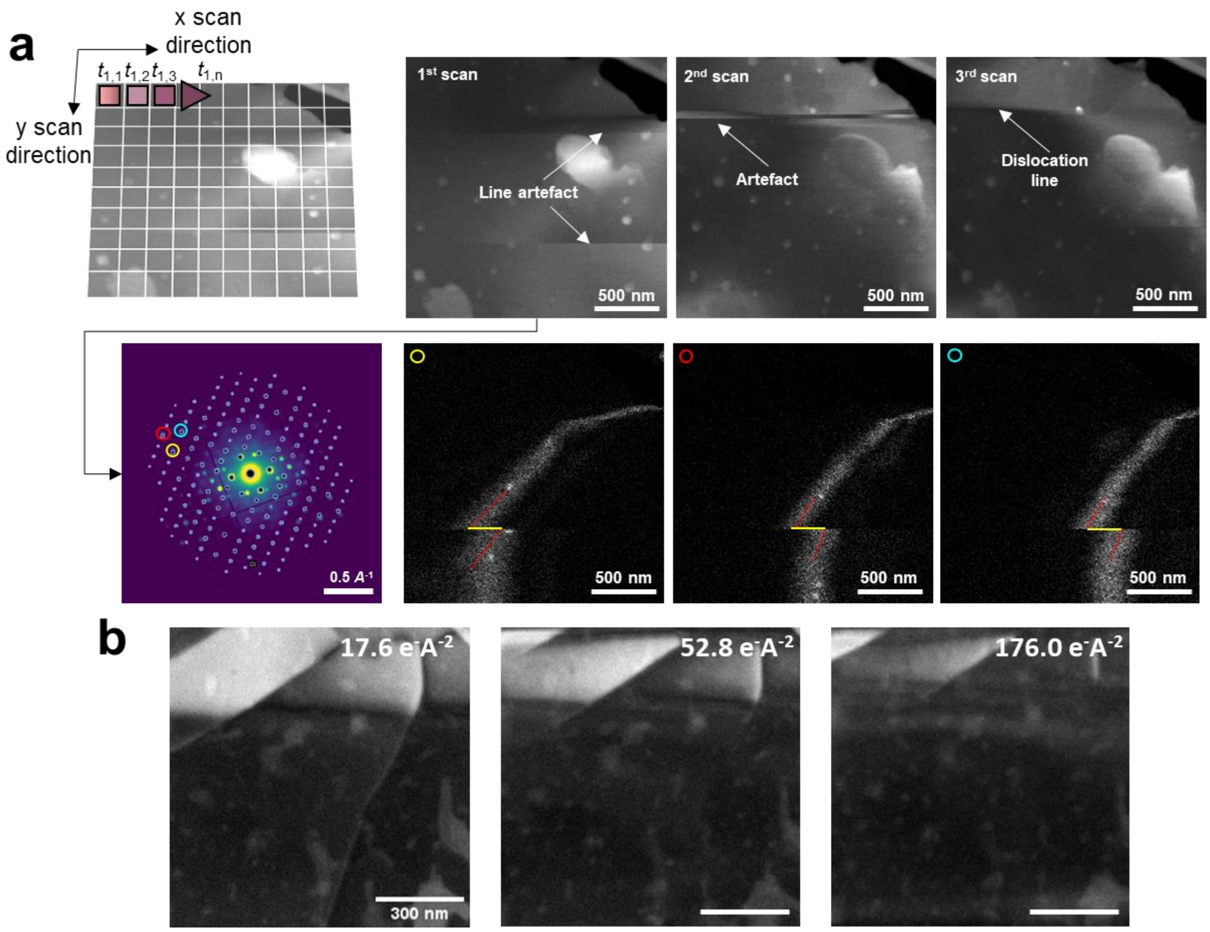

Supplementary Figure 28. Artefacts arising from SED due to the scanning of the electron beam on the sample for data sets taken at 200 kV and 300 kV, respectively. a, Series of ADF images from repeated SED measurement on the same area of p-terphenyl film showing the appearance of line artefacts parallel to the scanning direction of the electron beam (illustrated by the schematic – points are recorded sequentially in time and so the SED scan records a time-series of observations) which resemble dislocation lines. The line artefacts can be distinguished from true dislocations by their disappearance under repeated SED measurement. The displacement of the bend contours due to the line artefacts show no differences in magnitude at different diffraction vectors $\mathbf{g}_{hkl}$ that are not parallel, as marked in the diffraction pattern with on-zone simulated pattern overlaid in white. This finding can be used to distinguish line artefacts and true dislocations. In addition, an abrupt contrast change at the middle area between the 1st scan, 2nd scan, and 3rd scan indicates the movement of the bend contour, i.e. sample reorientation, at each scan. **b**, Series of ADF images from repeated SED measurement on the same area of anthracene film showing losses of crystallinity and dislocation contrast, as evidenced by the gradual disappearance of the line features and the bend contours when accumulated electron fluence increased. This observation demonstrates the scanning electron beam does not induce the formation of defect lines.

Supplementary Note 4: Measurements of beam damage

Critical fluence values are determined generally by examining the decay of a signal (e.g. diffraction intensity, image Fourier transform peak, spectroscopic signal) as a function of cumulative electron fluence. For a series of diffraction data acquired with constant exposure per unit time, the cumulative electron fluence can be calculated by multiplying the time that the samples had been exposed to the electron beam (in seconds) by the electron beam flux (J):

$$F(e^{-}\text{Å}^{-2}) = J \times (t_0 + t) \tag{S-16}$$

where t_0 is the time between the initial exposure of the analysed area to the electron beam and the time taken to record the image and the first diffraction pattern and t is the subsequent acquisition time of the following diffraction patterns. In this work, electron diffraction patterns were analysed by the custom-made Python scripts using functions from HyperSpy package (1.6.5)[9]. The spot intensity (I) of the selected diffraction spot was extracted from a series of diffraction pattern acquisitions to construct a time series, or equivalently, a cumulative fluence series. The damage of the samples is assumed to occur as an exponential decay of the spot intensity with decay rate τ as a function of cumulative electron fluence[10]:

$$I = I_0 \exp(-\tau F(t)) \tag{S-17}$$

Rearrangement to normalize by the initial intensity (I/I_0) for the particular selected spot establishes a linear relationship between $ln(I/I_0)$ and the cumulative fluence. Fitting this linear response enables determination of the gradient, with the critical fluence (CF) defined as $1/\tau$.

References

1. Jones, W. , Williams, J. O. Real space crystallography and defects in molecular crystals. *J Mater Sci*, **10**(3)**:** 379-386 (1975).
2. Newville, M., *et al.* lmfit/lmfit-py: 1.2.1 (1.2.1). (2023) doi:10.5281/zenodo.7887568.
3. Spiecker, E. & Jäger, W. Burgers vector analysis of large area misfit dislocation arrays from bend contour contrast in transmission electron microscope images. *J Phys Condens Matter*, **14**(48)**:** 12767 (2002).
4. Parsons, S. Introduction to twinning. *Acta Crystallographica Section D*, **59**(11)**:** 1995-2003 (2003).
5. Cherns, D., Preston, A. R. Convergent beam diffraction studies of interfaces, defects, and multilayers. *Journal of Electron Microscopy Technique*, **13**(2)**:** 111-122 (1989).
6. Wynne, E., *et al.* Grain and Domain Microstructure in Long Chain *N*-Alkane and *N*-Alkanol Wax Crystals. *Cryst. Growth Des.,* **24**(24): 10127-10142 (2024).
7. Bunn, C. W. The crystal structure of long-chain normal paraffin hydrocarbons. The "shape" of the >CH_2 group. *Trans. Faraday Soc.* **35**: 482-491 (1939).
8. Kawaguchi, A., *et al.* The Crystal Structure of Polyethylene at 4.5° K. *Bulletin of the Institute for Chemical Research, Kyoto University*, **55**(2): 217-226 (1977).
9. Peña, F., *et al.* hyperspy/hyperspy: Release v1.6.5. (2022) doi:10.5281/zenodo.5608741.
10. Ilett, M., *et al.* Analysis of complex, beam-sensitive materials by transmission electron microscopy and associated techniques. *Philosophical Transactions of the Royal Society A: Mathematical, Physical and Engineering Sciences*, **378**(2186)**:** 20190601 (2020).
