# Supplementary figures and images for: Microscopic crystallographic analysis of dislocations in molecular crystals
Source: Nat Mater. 2025 Mar 3;24(5):682–7. doi: 10.1038/s41563-025-02138-5 (PMC12048348; doi:10.1038/s41563-025-02138-5)

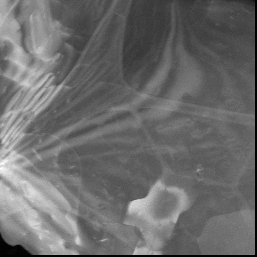

Supplement: Supplementary file 2 — Stacked images and diffraction patterns (Fig. 2a) for p-terphenyl and anthracene shown in Fig. 2b,c,f. [file 41563_2025_2138_MOESM2_ESM.tif]

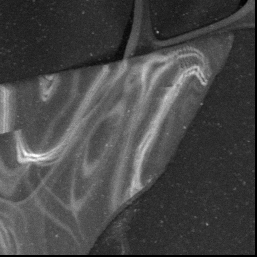

Supplement: Supplementary file 4 — Stacked images and diffraction patterns for theophylline and wax crystals shown in Fig. 3b,c,f,g. [file 41563_2025_2138_MOESM4_ESM.tif]

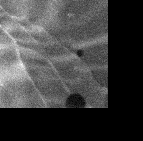

Supplement: Supplementary file 6 — Stacked images and diffraction patterns for p-terphenyl and theophylline crystals shown in Fig. 4. [file 41563_2025_2138_MOESM6_ESM.tif]
